# Allogeneic pASC transplantation in humanized pigs attenuates cardiac remodeling post-myocardial infarction

**DOI:** 10.1371/journal.pone.0176412

**Published:** 2017-04-27

**Authors:** Rafael Dariolli, Marcus V. Naghetini, Euclydes F. Marques, Celso K. Takimura, Leonardo S. Jensen, Bianca Kiers, Jeane M. Tsutsui, Wilson Mathias, Pedro A. Lemos Neto, Jose E. Krieger

**Affiliations:** Heart Institute (InCor), University of São Paulo Medical School, São Paulo, Brazil; Academia Sinica, TAIWAN

## Abstract

Cell therapy repair strategies using adult mesenchymal stromal cells have shown promising evidence to prevent cardiac deterioration in rodents even in the absence of robust differentiation of the cells into cardiomyocytes. We tested whether increasing doses of porcine adipose-tissue derived mesenchymal stem cells (pASCs) increase cardiac tissue perfusion in pigs post-myocardial infarction (MI) receiving angiotensin-converting-enzyme inhibitor (ACE inhibitors) and Beta-blockers similarly to patients. Female pigs were subjected to MI induction by sponge permanent occlusion of left circumflex coronary artery (LCx) generating approximately 10% of injured LV area with minimum hemodynamic impact. We assessed tissue perfusion by real time myocardial perfusion echocardiography (RTMPE) using commercial microbubbles before and following pASCs treatment. Four weeks after the occlusion of the left circumflex artery, we transplanted placebo or pASCs (1, 2 and 4x10^6^ cells/Kg BW) into the myocardium. The highest dose of pASCs increased myocardial vessel number and blood flow in the border (56% and 3.7-fold, respectively) and in the remote area (54% and 3.9-fold, respectively) while the non-perfused scar area decreased (up to 38%). We also found an increase of immature collagen fibers, although the increase in total tissue collagen and types I and III was similar in all groups. Our results provide evidence that pASCs-induced stimulation of tissue perfusion and accumulation of immature collagen fibers attenuates adverse remodeling post-MI beyond the normal beneficial effects associated with ACE inhibition and beta-blockade.

## Introduction

A loss of cardiomyocytes combined with fibroblast-derived collagen deposition alter the mechanical properties of the heart after a myocardial infarction (MI) and contributes to the functional deterioration of the organ and high cardiovascular morbidity and mortality [1–7]. Cell therapy regenerative strategies using embryonic stem cells (ESCs) [8] induced pluripotent stem cells (iPSCs) [9], and its derived cardiac progenitor cells (CPCs) [10] have all been explored, although with limited success. In contrast, cell therapy repair strategies using adult stromal cells from different tissue sources have shown promising pre-clinical evidence to prevent cardiac deterioration even in the absence of robust differentiation of the cells into cardiomyocytes. Thus, paracrine effectors acting over important healing pathways such as cell death, inflammation, and angio-vasculogenesis may be further explored as a pre-emptive therapeutic strategy [11–14], particularly in pre-overt ischemic heart failure conditions [14].

Consistent with this idea, tissue vessel density increases a few days after cell transplantation to support ischemic cardiac tissue, thereby further reducing cardiomyocyte death and tissue injury attenuating cardiac remodeling [11–13]. Scar formation during the healing process is markedly affected by local inflammatory factors ensued after the MI [15]. The amount of collagen and type of collagen fibers are directly related to the conversion of cardiac fibroblasts (CFs) into secretory myofibroblasts [16]. This conversion process can be modulated in vitro and in vivo by mesenchymal stromal cells therapy (MSC), altering the pattern of scar formation [17]. The amount of collagen and the level of maturation of the collagen fibers in the scar can be modulated by both the expression of adenylyl cyclase 6 (AC6) or the use of forskolin [18], suggesting that targeting these parameters may affect the final scar composition, which in turn will improve the mechanics of the pump and overall heart function post-MI [4]. In this context, several research groups are currently engaged in identifying the factors secreted by adult stromal cells to determine their targets and how the local ischemic microenvironment post-MI influences its secretion or how these factors act upon their targets. It is unknown whether we will succeed in replacing the secreted factors by small molecules and/or left with the challenge to increase retention and decrease the death of the transplanted adult stromal cells that would behave as the therapeutic source of growth factors. The latter poses pharmacological issues that have not been well resolved in terms of how the cells shall be harvested and manipulated, the best route of injection, number of cells or frequency of injections, etc.

Thus, in the present study, we aimed to establish the effective dose of allogeneic porcine adipose-derived mesenchymal stromal stem cells (pASC) to improve myocardial perfusion of immunocompetent pigs treated with daily doses of enalapril maleate and metoprolol succinate following an MI affecting approximately 10% of the left ventricle (LV) area and without detectable changes in cardiac function. Our findings showed that the highest dose of transplanted allogeneic pASC associated with drug therapy increased myocardial perfusion (MP) and vessel number, reduced the non-perfused fibrotic area, which presented predominantly immature collagen fibers, without cellular inflammatory response against pASC. Altogether, the cell transplantation was associated with an attenuation of the adverse remodeling of LV post-MI, suggesting that it may be further explored as a therapy strategy for cardiac repair or used as a pre-emptive strategy for non-overt ischemic heart failure.

## Results

### Intramyocardial allogeneic injection (IAI) of pASC increases myocardial perfusion post-MI

**Fig 1:**
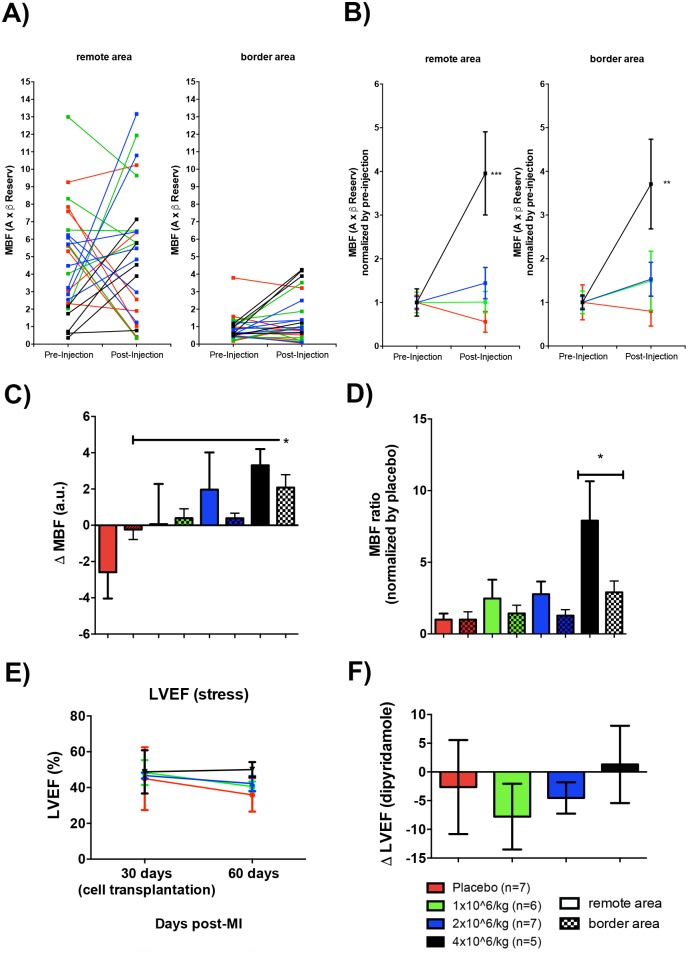
Myocardial perfusion increased after injection of 4 million cell/Kg. MBF was calculated by RTMPE. The highest dose of pASC increased myocardial blood flow (MBF) on both, remote and border areas of MI. A) MBF before and after cell injection for both non-infarcted (left chart) and infarcted (right chart) segments for each animal of the 4 experimental groups. B) MBF averaged value for each experimental group, normalized by the respective MBF averaged value before cell injection (difference are significant between 4 million cell/Kg injection animals before and after cell transplantation, *** p<0.0005 and ** p<0.005 to non-infarcted and infarcted segments respectively, Two-way ANOVA with Bonferroni post-test). C) ΔMBF from remote and border areas (ΔMBF = (MBF after cell injection)–(MBF before cell injection)), (differences are significant between 4 million cell/Kg injection and placebo groups in the infarcted segment, * p<0.05, One-way ANOVA with Bonferroni post-test). D) MBF ratio was calculated by averaged value for each experimental group, normalized by MBF average value of the placebo group (differences are significant between 4 million cell/Kg injection and placebo group (for each are separately), * p<0.05 for both remote and border area, One-way ANOVA with Bonferroni post-test). E) LVEF assessed by echocardiography and calculated from linear measurements before (30 days after MI induction) and 30 days after cells injections for each experimental group. F) ΔLVEF from percentage of LVEF after and before treatments (ΔLVEF = (LVEF after cell injection)–(LVEF before cell injection)). All data are means ± SEM.

Myocardial perfusion measured under dipyridamole stress, increased in the border and remote areas four weeks following intramyocardial injection of the highest dose of pASCs– 4 million cell/kg (Fig 1). The pre-injection perfusion values of all animals that received 4 million cell/Kg injections were increased or remained unchanged compared with the post-injection perfusion values, whereas the other experimental and the placebo groups showed inconsistent or deteriorating perfusion patterns in both assessed segments (Fig 1A and 1B). Interestingly, the experimental groups injected with 1 and 2 million cell/Kg showed a non-statistical trend to prevent the deterioration of myocardial blood flow (MBF) compared to the placebo group (Fig 1B and 1C). MBF ratio showed a significant increase in the border areas of the animals that received 4 million cell/Kg and a strong trend in the remote areas (Fig 1C). Altogether, normalized data showed a robust increase in MBF in both the remote and border areas, (approximately 6 and 3 times, respectively) compared with placebo animals (Fig 1D). Non-statistical trend to improvements were observed in the left ventricle ejection fraction (LVEF) in the animals treated with 4 million cell/Kg (Fig 1E and 1F).

### IAI of pASC increases the number of vessels in the border and remote areas of LV post-MI

**Fig 2:**
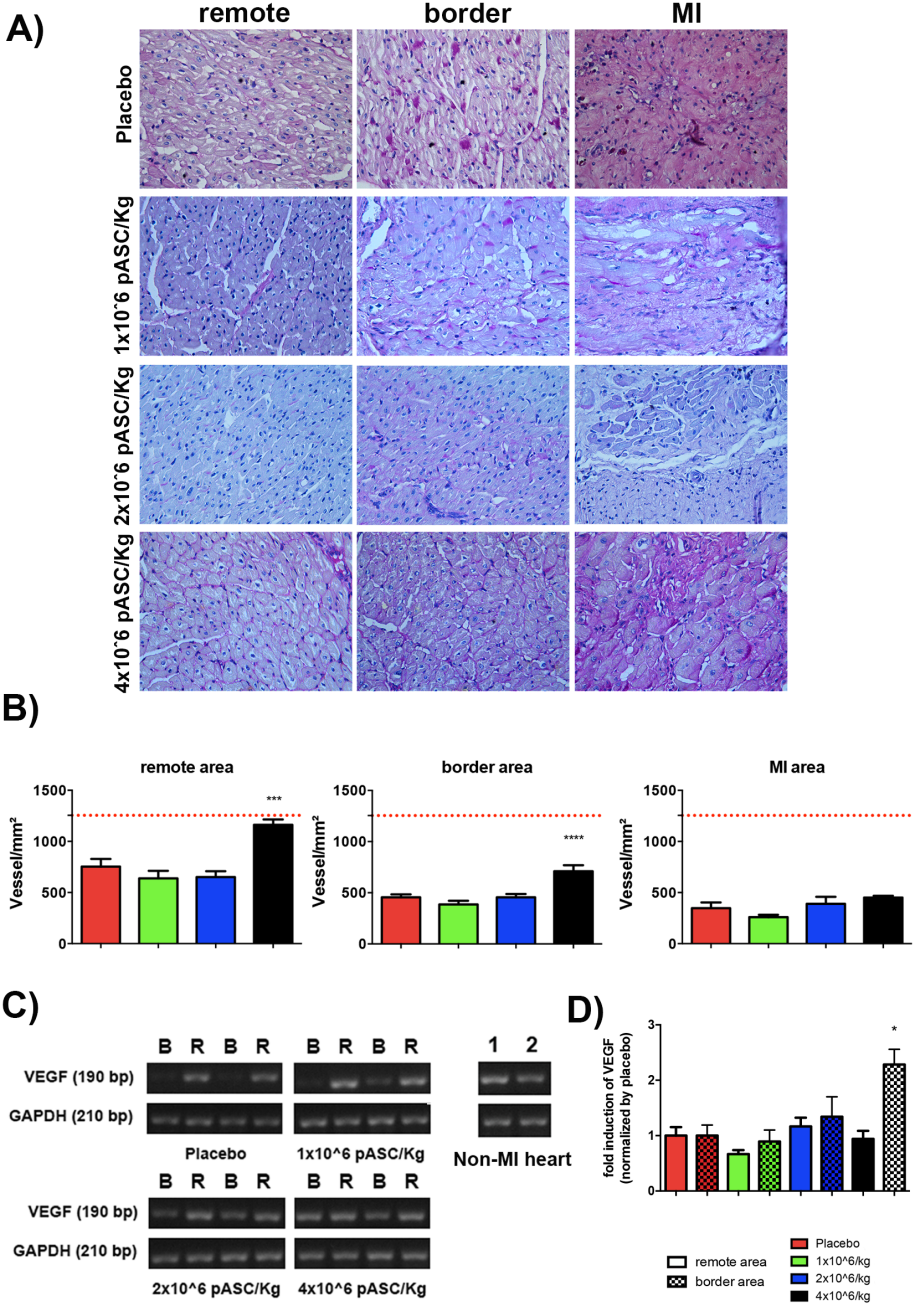
Injection of 4 million cell/Kg increased vascularization in border and remote areas of LV post-MI. Histological analyses of representative remote, border and MI areas of LV and subsequent quantification of vessels by manual counting (Periodic Acid Shift staining). A) representative images of stained by PAS per area and group. B) quantification of vessel number per area of tissue (in mm^2^) in remote, border and MI areas. The red dotted line represents the average number of vessels measured in LV of healthy pigs (N = 3). C and D) Semi-quantitative analysis of *VEGF* expression in border (B) and remote (R) areas of left ventricles for each experimental group. C) PCR products were loaded into agarose gels (two representative animals of each group are shown in the figure D) and band intensity was quantified and normalized by *GAPDH* expression (n = 4 animals per group). LV tissue from healthy pig was used as positive control. * p<0.05 compared to all the other experimental groups in different areas (ANOVA with Bonferroni post-test). Data are means ± SEM.

Ventricles transplanted with 4 million cell/Kg displayed higher vessel density than placebo and the other cell groups in both the border and remote areas (Fig 2A and 2B). In contrast, there was no increase in vascular density in the MI area of any of the experimental groups (Fig 2B). Moreover, we detected a two-fold increase in the expression levels of the angiogenic vascular endothelial growth factor (VEGF) in the border area of the 4 million cell/Kg animals, whereas the samples from the remote area of this group and the remaining LV samples from the experimental and placebo groups remained unchanged (Fig 2D and 2E).

### Effect of IAI of pASC on hemodynamics and LV morphometry post-MI

We have previously shown that closed chest permanent occlusion of the proximal LCx in pigs results in moderate MI injury (approximately 11% of the LV area) while overall cardiac function remains unchanged [19], specially when comparing to left anterior descending (LAD) occlusion models. In the present study, the injury assessed by echocardiography four weeks post-MI (pre-injection values) on the three experimental and placebo groups averaged 9.82 to 13.00% in the LV area. Importantly, all animals received daily Beta blocker (25 mg/day) and ACEi (10 mg/day) post-MI. Under these experimental conditions, the animals were clinically fit and presented no evidence of hemodynamic compromise under basal (Table C in S1 File) or stress conditions (Table 1) at baseline (30 days post-MI/pre-injection) compared to the experimental period (30 days post-injection) (Fig 1E and 1F and Figs B, C and D in S1 File).

**Table 1:** Pharmacological stress with dipyridamole echocardiographic linear measurements to assess cardiac function before and after cell injection. Data were represented by means ± SEM. P>0.05.

After Stress		Placebo (n = 7)		1x10^6^ pASC/Kg (n = 6)		2x10^6^ pASC/Kg (n = 7)		4x10^6^ pASC/Kg (n = 5)	
		Before injection	After injection	Before injection	After injection	Before injection	After injection	Before injection	After injection
Parameters		Mean±SEM	Mean ± SEM	Mean ± SEM	Mean ± SEM	Mean ± SEM	Mean ± SEM	Mean ± SEM	Mean ± SEM
MI area	(%)	10.08 ± 3.09	10.97 ± 4.02	10.27 ± 2.43	9.56 ± 2.62	10.22 ± 1.62	8.37 ± 1.34	12.51 ± 1.45	7.77 ± 1.16
Septum	(mm)	0.51 ± 0.11	0.58 ± 0.11	0.53 ± 0.10	0.61 ± 0.10	0.51 ± 0.08	0.54 ± 0.13	0.65 ± 0.17	0.51 ± 0.01
PW	(mm)	0.53 ± 0.18	0.41 ± 0.09	0.34 ± 0.05	0.43 ± 0.05	0.38 ± 0.02	0.38 ± 0.04	0.47 ± 0.15	0.46 ± 0.04
EDD	(mm)	4.14 ± 0.33	4.34 ± 0.32	4.16 ± 0.29	4.57 ± 0.51	4.44 ± 0.29	4.48 ± 0.65	4.16 ± 0.60	4.24 ± 0.57
ESD	(mm)	3.21 ± 0.60	3.60 ± 0.34	3.13 ± 0.44	3.66 ± 0.36	3.41 ± 0.32	3.57 ± 0.67	3.14 ± 0.60	3.17 ± 0.43
LVSF	(%)	22.92 ± 10.20	17.24 ± 4.79	24.88 ± 9.51	19.82 ± 6.31	23.28 ± 2.43	20.84 ± 5.79	24.64 ± 6.46	25.17 ± 2.34
EDV	(cm^3^)	76.83 ± 13.98	85.80 ± 15.65	77.40 ± 13.06	96.09 ± 29.69	90.19 ± 13.38	94.25 ± 30.86	79.26 ± 24.49	82.62 ± 25.07
ESV	(cm^3^)	43.66 ± 17.58	55.01 ± 12.00	39.92 ± 13.06	56.86 ± 15.26	48.59 ± 10.65	56.16 ± 23.13	41.59 ± 20.27	41.26 ± 12.70
SV	(cm^3^)	33.17 ± 9.34	30.79 ± 8.57	37.48 ± 13.87	39.23 ± 21.09	41.60 ± 2.84	38.10 ± 12.83	37.67 ± 11.40	41.36 ± 12.92
LVEF	(%)	44.96 ± 16.00	35.90 ± 8.63	48.36 ± 15.57	40.58 ± 10.61	46.73 ± 4.22	42.20 ± 10.34	48.73 ± 10.83	50.03 ± 3.76
LV Penn Mass	(g)	58.70 ± 20.60	59.23 ± 15.98	48.06 ± 13.94	68.80 ± 28.80	53.99 ± 11.08	59.05 ± 19.89	67.03 ± 24.94	55.88 ± 13.54

MI: myocardial infarction, PW: posterior wall, EDD: end-diastolic diameter, ESD: end-systolic diameter, LVSF: left ventricle shortening fraction, EDV: end-diastolic volume, ESV: end-systolic volume, SV: stroke volume, LVEF: left ventricle ejection fraction, LV: left ventricle.

**Fig 3:**
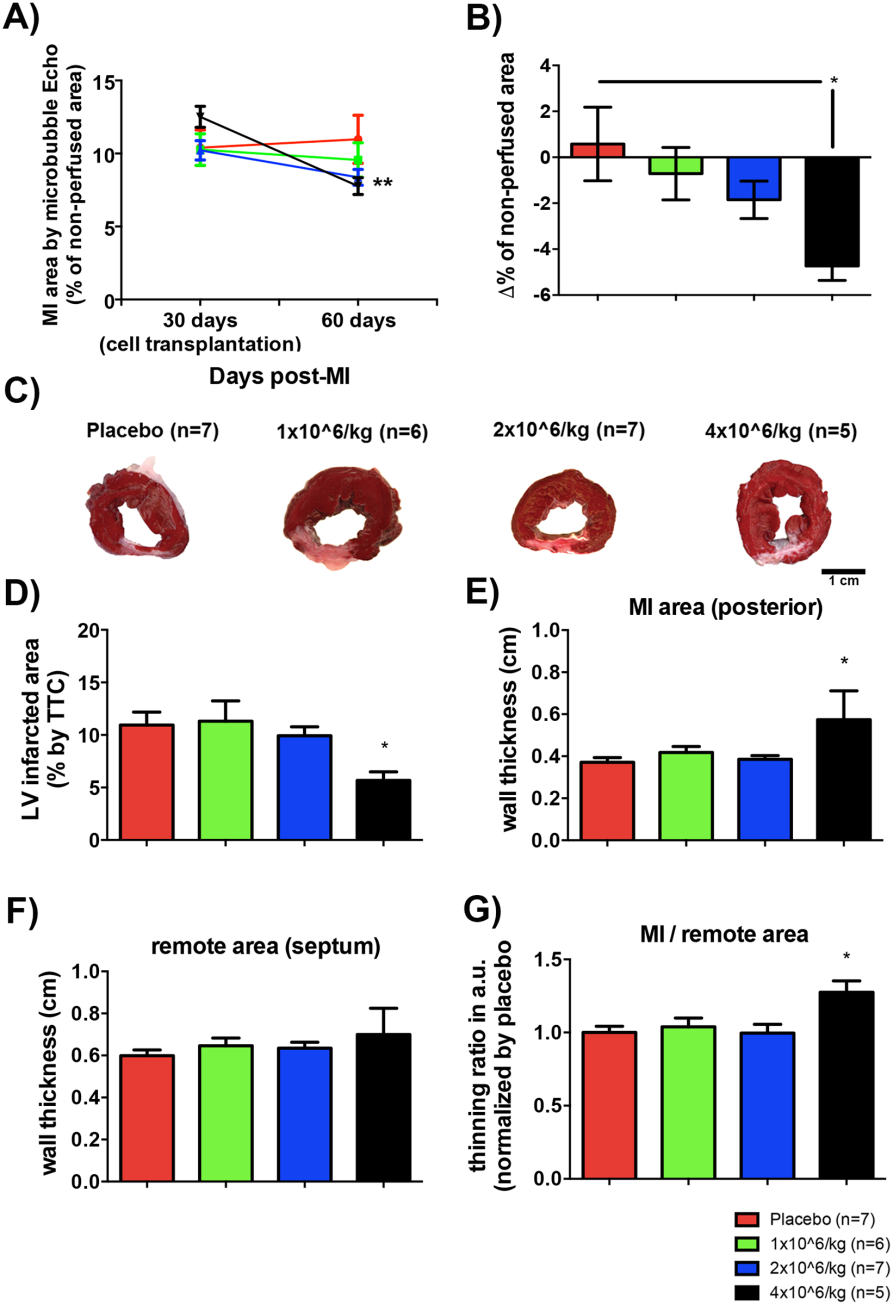
Injection of 4 million cell/Kg reduced left ventricle injured area. A) Percentage of MI (non-perfused area of LV) before and after pASC injection quantified by planimetry using micro-bubble contrasting images (differences are significant 4 million cell/Kg comparing time, ** p<0.005, Student’s t-test). B) Δ% of non-perfused area for the four experimental groups (Δ% = (% after cell injection)–(% before cell injection) (difference is significant between 4 million cell/Kg injection and placebo group * p<0.05, One-way ANOVA with Bonferroni post-test). C) Representative papillary level slices after TTC stain. D) Averaged percentage of infarcted area for each experimental group (difference is significant between 4 million cell/Kg injection and placebo group * p<0.05, One-way ANOVA with Bonferroni post-test). E) Averaged thicknesses of posterior (MI) wall for each experimental group (difference is significant between 4x10^6 cell injection and placebo group * p<0.05, One-way ANOVA with Bonferroni post-test). F) Averaged thicknesses of septum (remote) wall for each experimental group. G) Thinning ratio (MI wall thickness/remote wall thickness) normalized by thinning ratio of the placebo group (difference is significant between 4 million cell/Kg injection and placebo group * p<0.05, One-way ANOVA with Bonferroni post-test). Data are means ± SEM.

Using the contrasting microbubble agent, we defined the non-perfused area of LV before and after pASC injections. The 4 million cell/Kg dose injected in LV showed a 38% reduction of the non-perfused area 30 days after cell injection compared with the placebo group (Fig 3A and 3B). Interestingly, pigs that received 1 million and 2 million cell/Kg showed a slight trend toward non-perfused area reduction (Fig 3A and 3B). Consistent with the echocardiographic assessments, thirty days after injection of the highest pASC dose, we observed a significant decrease in the scar area compared with the other experimental and placebo groups (Fig 3C and 3D). We indexed the data by body weight and normalized the data against the placebo group. We found no differential LV hypertrophy among the groups (Fig E in S1 File). Furthermore, MI adverse remodeling was attenuated in the highest pASC dose as shown by a decrease in MI wall thinning (Fig 3E) and no wall septum hypertrophy (Fig 3F), which resulted in an increase of the thinning ratio in in the group 4 million cell/Kg compared to the other groups (Fig 3G).

### IAI of pASC increased the deposition of immature fibers post-MI

**Fig 4:**
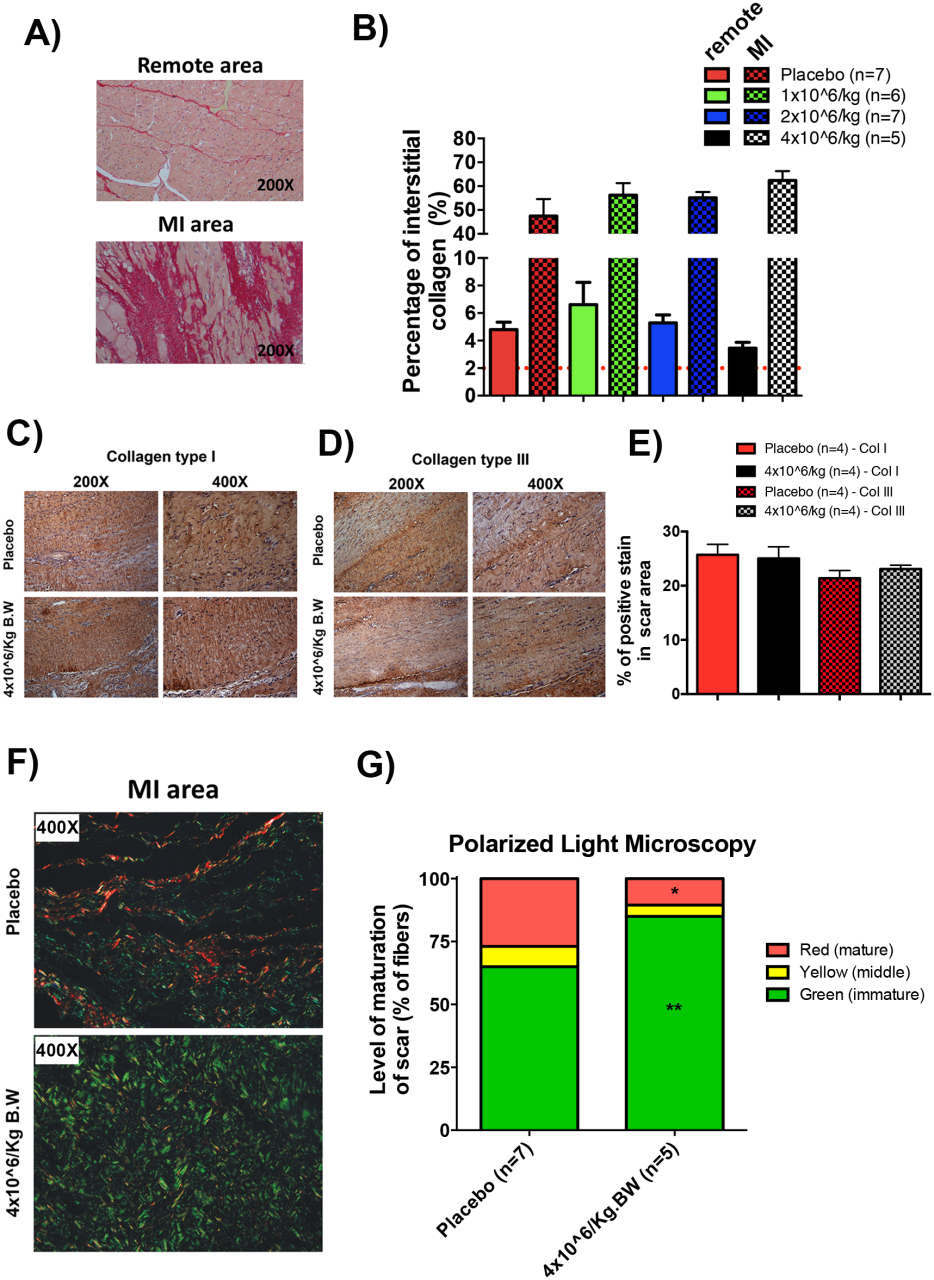
Injection of 4 million cell/Kg increased the deposition of immature fibers in the scar post-MI. A) Representative images of septum (remote area) and posterior wall (MI-border transition area) of left ventricle stained with PicroSirius-red. B) quantification of interstitial collagen in the septum (remote area) and posterior wall (MI-border transition area). C) representative immunohistochemistry images of MI scar area, showing staining of collagen fibers types I D) and type III, in placebo and 4 million cell/Kg injection groups and E) quantification of the percentage of positive stain of collagen types for both group. F) representative polarized light microscopy (non-antibody reaction) images of scar areas showing collagen mature fibers in red and immature fibers in green for both placebo and 4 million cell/Kg injection groups and G) quantification of collagen fiber maturity in scar area for both placebo and 4 million cell/Kg injection groups (differences are significant between 4 million cell/Kg injection and placebo group * p<0.05 to mature (red) fibers and ** p<0.005 to immature (green) fibers, Student’s t-test to each maturity fibers). Data are mean ± SEM.

Total interstitial collagen deposition increased in similar levels in both the remote and MI areas in the experimental and placebo groups (Fig 4A and 4B). Similarly, the ratio of Collagen types I and type III remained unchanged (approximately 1:1) between 4 million cell/Kg dose and placebo groups (Fig 4C–4E). Despite this finding, we found increased deposition of immature fibers of collagen in group 4 million cell/Kg compared to the placebo group using polarized light microscopy. Thus, the placebo hearts displayed a pattern characterized by mature fibers (Fig 4F, red staining and 4G quantification), which is known to confer greater stiffness to the LV. Additionally, non-important differences in collagen maturity in remote areas were shown in Fig F in S1 File.

### IAI of pASCs do not elicit increased cellular inflammatory response in the heart

**Fig 5:**
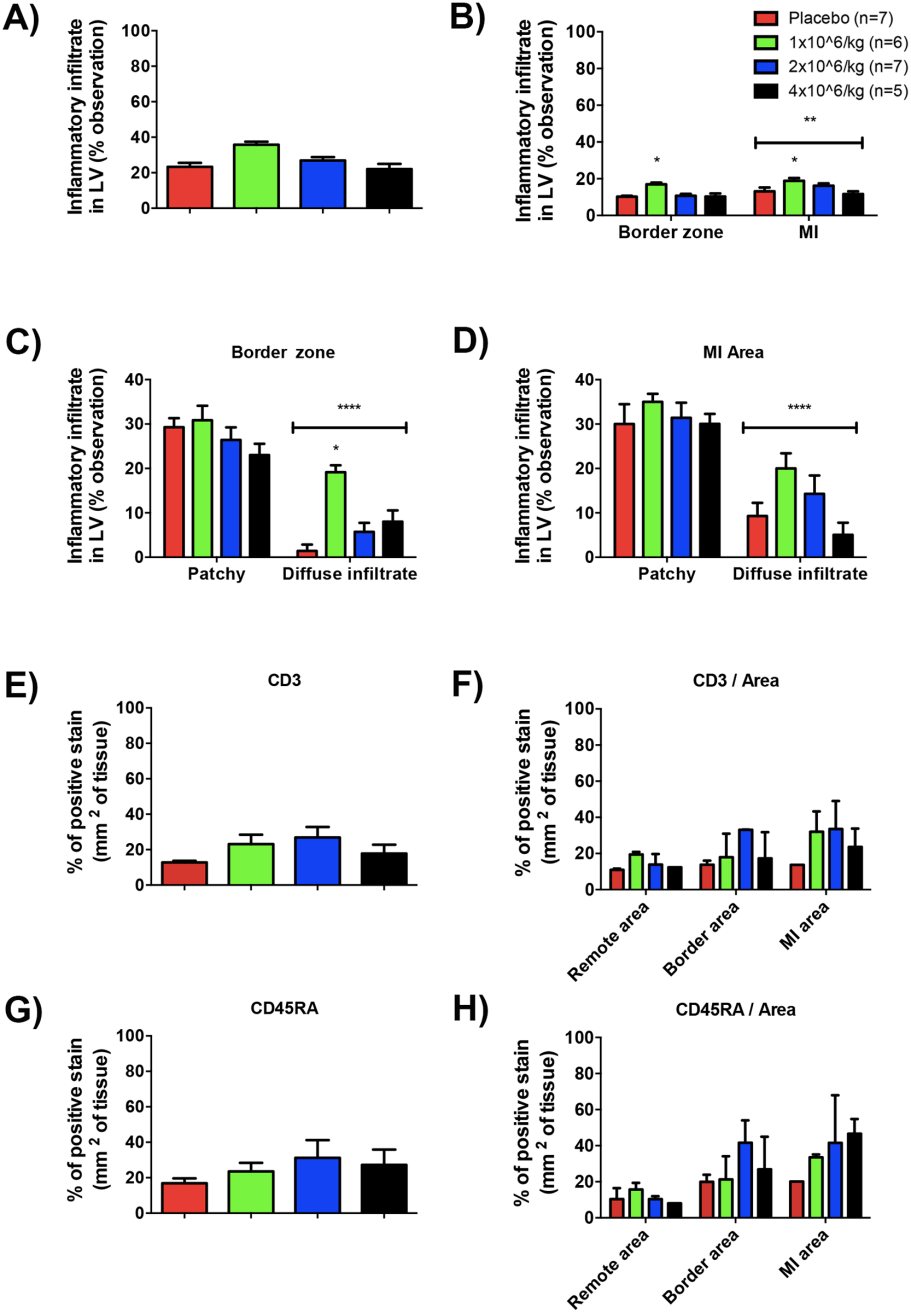
Injection of 4 million cell/Kg did not trigger increased cellular inflammatory response into the heart. Based on, histological slices stained with H&E and an observational method (grade score) the inflammatory profile of infarcted LV tissue was assessed. A) Frequency of total inflammatory cells observed inside LV muscle for all experimental groups (difference is significant between 1 million cell/Kg injection vs. placebo, 2 and 4 million cell/Kg groups; ** p<0.005, One-way ANOVA with Bonferroni post-test). B) Frequency of inflammatory cells ***per area*** observed inside LV muscle for all experimental groups (difference is significant between 1 million cell/Kg injection vs. placebo, 2 and 4 million cell/Kg groups on both areas (border and MI); * p<0.05 and MI vs. border area, ** p<0.005 (area effect), Two-way ANOVA with Bonferroni post-test). C) Frequency of inflammatory cells ***per type*** observed inside the ***border area*** of LV muscle for all experimental groups (difference is significant between 1 million cell/Kg injection vs. placebo, 2 and 4 million cell/Kg groups; * p<0.05 and diffuse vs. patchy infiltration, **** p<0.0001 (type of infiltrated cells effect), Two-way ANOVA with Bonferroni post-test). D) Frequency of inflammatory cells ***per type*** observed inside the ***MI area*** of LV muscle for all experimental groups (difference is significant between diffuse vs. patchy infiltration, **** p<0.0001 (type of infiltrated cells effect), Two-way ANOVA with Bonferroni post-test). To improve quality of the observation, antibody-specific immunohistochemistry reactions against CD3 and CD45RA lymphocyte markers were performed. E) Quantification of CD3-positive cells by automatic counting to measure the percentage of positive stain on representative immunohistochemistry slides for all experimental groups. F) Quantification of CD3-positive cells per area of LV tissue. G) Quantification of CD45RA-positive cells. H) Quantification of CD45RA-positive cells per area of LV tissue. Data are mean ± SEM.

Pathological analyses were performed in the LV tissue by Hematoxylin & Eosin (H&E) staining to assess cellular inflammatory response 30 days after pASC injections. The doses of injected cells did not elicit an increase in inflammatory infiltrated cells (Fig 5A, 5E and 5G). As expected, the inflammatory cells were observed only in the border and MI (Fig 5B–5D and Table D in S1 File). Despite the non-statistical difference observed in the total heart frequency of inflammatory infiltration, animals from 1 million cell/Kg group showed a significant higher amount of inflammatory cells compared to other experimental groups and placebo (Fig 5B). As expected, MI areas showed significantly higher infiltrate than border areas for all the groups (Fig 5B). Inflammatory responses were also qualified by type. Patchy and diffuse infiltrates were observed in both areas (border and MI) in all the groups. The frequency of diffuse infiltrate was higher in 1 million cell/Kg group compared to other groups but not in patchy ones (Fig 5C). No differences were observed in the frequency of patchy or diffuse infiltrates between experimental or placebo groups in MI areas (Fig 5D). Further, diffuse infiltrates were lower than patchy ones on both, border and MI areas (Fig 5C and 5D). In addition, immunohistochemical analysis of CD3 and CD45RA lymphocyte populations reinforced a non-significant difference of inflammatory infiltrates on both total area of LV (Fig 5E and 5G) and isolated areas (Fig 5F and 5H) in experimental or placebo groups.

### There is no evidence for the transplanted allogeneic pASC into the cardiac tissue of recipient pigs

**Fig 6:**
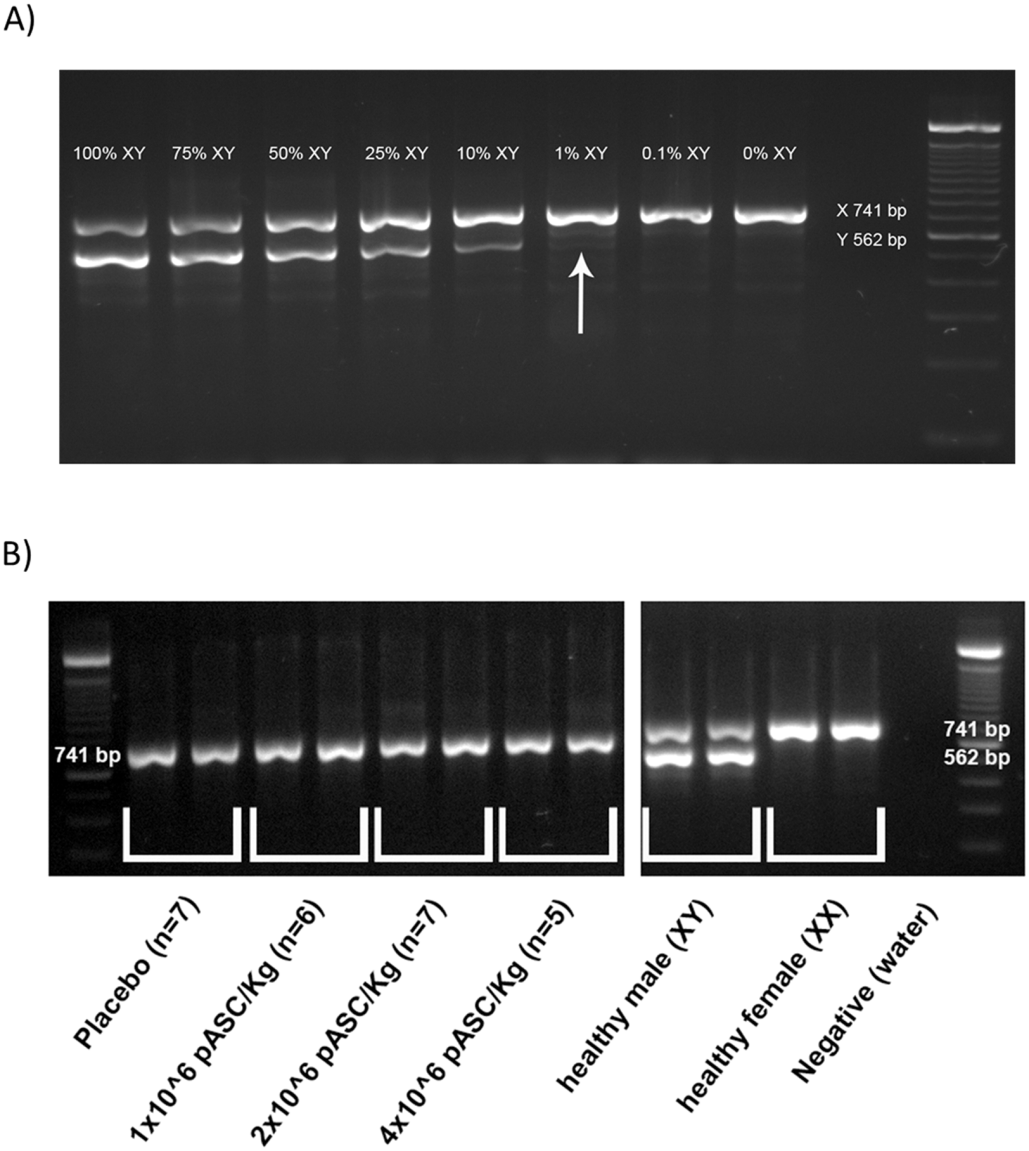
pASCs can not be found on the host tissue 30 days after injection. A) PCR-based assay to specifically amplify a Y-chromosome segment in different mix of male/female genomic DNA pools, leading to 741 and 562 bp amplicons for X and Y chromosome, respectively. It is possible to identify up to 1% of male DNA (white arrow). B) PCR-based assay applied on genomic DNA extracted from border zone of infarcted hearts of the four experimental groups (injection site). Note that no signs of Y chromosome fragment of DNA amplification (male injected cells) can be observed into the female “host” tissue samples.

To identify the transplanted cells 30 days after injection, male donor cells were injected into female infarcted hearts, and the detection of a length polymorphism in the sexual chromosomal amelogenin gene was used as a marker of the Y chromosome. Semi-quantitative PCR was performed to detect differences in the PCR product of X and Y chromosomes. The method was standardized using a mixture of male and female DNA (Fig 6). Despite the capacity to identify 1% of male DNA mixed with 99% of female DNA (Fig 6A), no traces of male DNA were measured into female LV tissue 30 days after cell injection (Fig 6B). Furthermore, we found no teratomas or teratocarcinoma in pigs after the allogeneic injection of pASCs.

## Discussion

The main finding of the present study is that the highest dose of transplanted adipose-derived mesenchymal stromal cells (ASC) into the myocardium (4 million cell/Kg) attenuated the adverse remodeling of the LV by promoting an increase in vessel density and tissue perfusion accompanied by a pattern of tissue collagen deposition with a predominance of more immature collagen fibers and reduction in scar size regardless of the absence of transplanted cells 30 days after injection.

Cell therapy has been considered a promising adjunct/replacement approach for the treatment of ischemic heart diseases [12,13,20], but several issues remain unclear, including basic pharmacological considerations, such as cell number administered, preferable route, and frequency of cell administration. Previous data from our lab showed that only 5–7% of bone-marrow (BM) MSC [13] and ASC [12] remain in the heart of rats 24 hours after a trans-epicardial injection while alternative routes resulted in virtually no cardiac engraftment. The delivery of cells combined with biopolymers enhanced the cardiac engraftment of transplanted cells 2-5-fold [12,13], highlighting that the efficiency of these procedures is limited, particularly if one anticipates a close correlation between functional improvement and the number of engrafted cells into the LV. In the pig model, *Poncelet et al*. demonstrated that injecting 1 million cell/Kg 2 weeks after MI increased the capillary density in the viable myocardium [21], whereas *Quevedo et al*. showed vessel formation and myocardial blood flow improvement by injecting 200 million cells 20 weeks after MI [22]. The findings of the present study using quantitative and functional RTMPE provide further support to these observations and suggest that these effects on MBF and vessel formation are related to the dose of pASC (4 million cell/Kg) transplanted. This is a standard method to assess myocardial perfusion for both humans [23,24] and animal models [25,26]. RTMCE is a versatile method to detect contrast microbubbles at the capillary level in the myocardium to assess tissue viability. It displays an equivalent sensitivity compared with SPECT in microvascular disease and coronary artery disease (CAD) diagnosis[23,24,27–29]. It is important to highlight that potential limitations of the method in the clinical setting can be easily controlled in the pre-clinical scenario [23–29].

The pre-clinical evidence indicates that cardiac function improvement is weakly linked to the transdifferentiation of mesenchymal stromal cells into cardiomyocytes or endothelial cells [12,13,30–32]. Alternatively, the underlying mechanisms associated with prevention of cardiac deterioration are most likely mediated by the paracrine action of released bioactive molecules, resulting in a pleiotropic response affecting a number of biological processes related to inflammation, cell death, angio-vasculogenesis, and matrix remodeling [11–13]. Adult mesenchymal stromal cells are known to produce and secrete a large number of cytokines, and presumably the local MI microenvironment modulates their behavior and/or the interaction of the secreted cytokines with their targets under this unique microenvironment (low O_2_, high reactive oxygen species and increased strain). During cardiac ischemia, a series of vascular cytokines, such as VEGF, fibroblast growth factor beta (βFGF), Insulin-like growth factor (IGF-1), Platelet Derived Growth Factors (PDGF), and angiopoietin are required to activate vascular reparative pathways [33–36]. In the present study, we documented important changes including an increased density of vessels and blood tissue perfusion associated with a smaller scar that is rich in immature collagen fibers 30 days after cell transplantation. We found no evidence for the presence of the transplanted cells after this period, which could explain, at least in part, the absence of differential tissue cellular inflammation in response to the allogeneic injection; it also highlights the opportunity to devise novel strategies to enhance cell retention/survival to achieve greater therapeutic efficiency for long time periods. Others have showed sparse number of cells in the myocardium weeks after transplantation also indicating that robust transdifferentiation is unlikely and pointing towards a scenario where secreted factors act in concert to produce a pleiotropic beneficial response to prevent or delay cardiac remodeling post-MI [37,38].

There were several evidences indicating that the animals transplanted with the highest dose of cells presented a smaller scar and increased perfusion, which included echocardiographic planimetry, the determined non-perfused areas of the LV and post-mortem assessment of scar area and thickness of the affected posterior wall. The progression of MI is related to the replacement of necrotic tissue for fibrosis, resulting in disorganization of the extracellular matrix (ECM) [39]. This morphological alteration affects the mechanics of the ventricular contractile function, altering the normal heart elliptical shape towards a spherical form that will result in alterations of end systolic volume index [40]. Epidemiological evidence indicates that prevention of wall thinning is associated with better prognostic after MI [41,42]. *Dai and colleagues* showed that increasing the scar thickness by injecting collagen mixed solution (95% of col. I and 5% of col. III) improves LV stroke volume and ejection fraction and prevents paradoxical systolic bulging post-MI in rats [39]. The implant of an injectable absorbable alginate scaffolds is another acellular approach that prevents scar thinning, adverse LV remodeling and dysfunction in early (7 days) and late (60 days) post-MI [43,44].

In rats, injection of bone-marrow or adipose-derived mesenchymal stromal cells on the border of the MI attenuates wall thinning and changes the composition of fibrotic tissue in the injured wall of LV [12,13]. In the pig model used in this study, the scar size was approximately 10–11%, which is comparable to human MI, whereas in the rat model, the MI scar is much larger, reaching 35–45% of the LV. Thus, it is important to emphasize that we found similar higher total tissue collagen and collagen types I and III in all groups, except in group 4, where the collagen fibers became predominantly more immature. This finding may be significant, and we speculated that the reduction in the scar area in the highest dosage cell group is a consequence of combining a more elastic (less stiff) scar with an increased perfusion in the adjacent tissue, providing increased mechanical performance to the ventricle. This idea must be pursued in the future because it would imply that the scar is plastic even 30 days post-MI when the cells were transplanted. This finding opens an interesting and hitherto unexplored window for intervention post-MI that presently has only been only targeted pharmacologically.

Consistent with our finding of the lack of tissue inflammatory cellular response associated with allogeneic transplantation, adult MSCs have been shown to present low immunogenicity in allogeneic treatments *in vivo* [21,22,45–47] and are capable of suppressing lymphocyte proliferation *in vitro* [45,46,48]. This issue has recently being the subject of a meta-analysis demonstrating the similar effects of autologous and allogeneic therapy in ischemic heart disease in large animal models [49]. Alternatively, one may consider that there was a response that was resolved by day 30, particularly considering that we found no evidence for the presence of the transplanted cells after 30 days. If the former is the predominant explanation, then it raises the interesting possibility of having *off-the-shelf* allogeneic therapeutic cells ready for MI patients.

Altogether, we provide evidence that ASC transplantation prevents cardiac remodeling post-MI via a dose-dependent increase in tissue perfusion and scar reduction with collagen tissue deposition with a predominance of immature fibers. This is consistent with the idea that pASC transplantation may give rise to a more elastic and small scar contributing to improve cardiac mechanics and ventricle performance post-MI beyond the normal beneficial effects associated with ACE inhibition and beta-blockade, present in all groups regardless of the absence of transplanted cells 30 days after injection.

## Material and methods

### Study design

Different doses of allogeneic pASCs from a male donor were transplanted directly into the LV border area (electrically less competent than remote area but more competent than MI scar) of MI in pigs treated with enalapril maleate and metoprolol succinate to verify whether changes in the extracellular matrix and augmentation of tissue vessel density and perfusion minimized the deleterious effects of MI. All experiments were conducted per the protocol approved by the Institutional CAPPesq Ethics Committee (protocol number #022/09). Animal care complied with the ARRIVE guidelines (Animals in Research: Reporting *In Vivo* Experiments) [50]. The animals included in the study were blinded randomized in the experimental groups. Forty female pigs (*Sus scrofa domestica*, MS60 EMBRAPA—weight, 15 to 20 kg) were used in this study. All animals were subjected to clinical examinations before the protocol, and only healthy animals with no signs of diseases were included. Pigs were maintained in a local commercial swine farm (Granja RG, Suzano-SP, Brazil) with free access to food and water. For acclimatization, the animals were brought to the experimental facility 24–72 hours before experimentation. The anesthetic procedures applied were previously established by *Dariolli et al*.[19].

***The experimental design*** consisted of three phases (Fig A in S1 File). In ***phase I***, forty pigs were subjected to baseline angiography followed by MI induction. Five animals died during phase I due to MI complications. Twenty-four hours after MI induction, the animals were subjected to a continuous treatment with 10 mg of enalapril maleate and 25 mg of metoprolol succinate once daily. Thus, in ***phase II***, thirty days after MI induction, a baseline morphological and functional echocardiogram was performed in all animals, and only animals with similar MI (size and function) were included and blindly randomized into 4 groups to receive different doses of pASC in the border of the infarction (PBS control group, 1, 2 or 4 million of cells (pASCs) per kilogram of body weight, respectively). An additional control group of 6 animals had the MI induced, but did not receive any treatment (cellular or drug treatment—control of survival). Three pigs died during echocardiogram examination due to MI complications (allergic reaction against micro-bubble contrast; 1 pig from 1 million cell/Kg and 2 pigs from 4 million cell/Kg). In ***phase III***, thirty days after cell injection, the pigs underwent a new echocardiography, angiographic assessment and were then euthanized with high doses of sodium thiopental followed by an overdose of potassium chloride. During phase III, only one pig died due to MI complication (irreversible ventricular fibrillation 15 days after cell injection; group 1 million cell/Kg).

All the data showed in this article were obtained from the animals that completed the therapeutic cell protocol (Fig A(B) in S1 File; 7, 6, 7 and 5 viable pigs in each group, respectively).

### pASC isolation, characterization, and stromal cell bank generation

After anesthesia and asepsis procedures, subcutaneous adipose tissue was extracted by surgical procedures from one adult male pig. pASCs were isolated and characterized, and a stromal cell bank was generated as previous described by *Dariolli et al*.[51]. Briefly, pASCs were isolated from 300g of adipose tissue by enzymatic digestion of fat (collagenase type 1A 0.075%), cultured in Dulbecco's Modified Eagle's Medium-Low glucose (DMEM-Low) supplemented with 10% fetal bovine serum (FBS) until passage 4 and then frozen in vials with 3.3 billion of cells in 10% of Dimethyl sulfoxide (DMSO) solution. The appropriate number of vials was thawed no more than 30 minutes before the injection procedures. Cells were washed twice in sterile Phosphate-buffered saline (PBS) and suspended in 4 mL sterile PBS to posterior injections.

### Percutaneous model of myocardial infarction

A catheter-based model of MI by permanent occlusion of the left circumflex coronary artery (LCx) using foam-sponge as trombogenic material was used as previously described by *Dariolli et al*. [19]. Briefly, after anesthetic and aseptic/antiseptic procedures, a 0.014 guidewire was introduced into the distal portion of LCx (Merit Medical’s). Pieces of foam sponge were placed into the proximal portion of LCx, using the guidewire and a balloon catheter, causing an embolism. An angiographic image was performed 5–10 min after sponge placement to confirm the success of the obstruction.

### pASC injection

Thirty days after MI induction, the animals underwent thoracotomy in the left fifth intercostal space. Pericardium was visualized and excised. The posterior and lateral walls of the LV were visualized, and an ***“epicardial electrical mapping”*** of the injury was then performed (more details are the in Supporting material). The electrical mapping result was associated with an echocardiographic morphological evaluation to delimit the border of MI. So, pASCs (1, 2 or 4 million cell/Kg) were suspended in 4 mL of sterile PBS and 200uL injections were performed in 20 different sites around the border of MI (Fig A(C) in S1 File). Insulin syringes (0.5 mL, 29G –BD Ultra-Fine^™^) were used to perform cell injections. All the procedure was performed by a surgeon who was blinded to the experimental groups. After injection, the incisions were sutured, and the air inside the pleural cavity was removed.

### Echocardiographic assessments

The echocardiographic assessments of perfusion was performed based on well stablished and published protocols adjusted to animal models [25,26]. In brief, a commercially available ultrasound system (Sonos 7500—Philips Medical Systems, Bothell, Washington, USA) were used to acquire the echocardiographic images. ***Linear measurements*** were obtained in parasternal long-axis images. LV volumes were determined using the *Teichholz* formula. The end-diastolic and end-systolic areas of LV were measured by planimetry in parasternal-papillary short-axis images. ***Real time myocardial perfusion echocardiography (RTMPE)*** imaging was performed in the parasternal long and short-axis using power modulation mode with a mechanical index of 0.2, a frame rate 25–30 Hz, and continuous intravenous infusion of lipid-encapsulated microbubbles Sonovue^®^ (Bracco, Milan, Italy). Microbubble destruction was achieved using a packet of five high-intensity pulses. Low-power perfusion images containing 15 cardiac cycles were acquired. After baseline acquirement, hyperemia was induced by dipyridamole infusion via a parallel port. A total dose of 0.56 mg/Kg of dipyridamole was infused during 4 minutes. At the end of dipyridamole infusion, a second acquisition of images was performed. ***Quantitative Analysis of RTMPE*** was performed by off-line image analysis using commercially available software (Q-lab 6.0 Phillips Medical Systems, Bothell, WA, USA). Myocardial blood flow was quantified in the infarcted areas, border zone (approx. 10–20 millimeters out of MI scar) and normal areas. Myocardial plateau (A_M_, dB) and adjacent left ventricular plateau (A_LV_, dB) signal intensities and signal intensity exchange rate (ß, s^-1^) were calculated automatically using the software. Normalization to myocardial acoustic intensity (An) was defined as the ratio of A_M_ divided by A_LV_ and the index of blood flow was defined as the product of An and ß (s-1) (more details are in the Supporting material). ***Myocardial non-perfused percentage*** was assessed by planimetry of non-perfused tissue in parasternal-papillary short-axis images obtained after pharmacological stress with dipyridamole protocol, these images were quantified using *ImageJ* software. Echocardiographic images and analyses were performed by an expert who was blinded to the experimental groups.

### Anatomopathological analyses

After terminal functional assessment by echocardiogram and angiography, the animals that were still under anesthesia were killed by an overdose of potassium hydrochloride (KCl). After cardiac arrest, the heart was removed and dissected to obtain the LV as previously described by *Dariolli et al*. [19]. Briefly, LV was sectioned transversely from the base to apex in 5-mm sections (on average 7 sections, Fig A(D) in S1 File) and processed with specific stain techniques. All the measurements were performed for two trained operators who were blinded to the experimental groups.

#### Macroscopic assessment

LV sections were stained with 2,3,5 Triphenyl tetrazolium chloride (TTC) for macroscopical assessments. ***The area of necrotic tissue*** was measured by planimetry. The ***wall thickness*** of papillary-level section was measured in triplicate (Fig A in S1 File). The ***thinning ratio*** was determined as the ratio of averages of remote wall thickness and injured wall thickness. All the measurements were obtained using *ImageJ* software [52].

#### Microscopic assessment

LV section was divided into three segments: remote, border zone (approx. 10–20 mm out of the MI scar) and injured area. These segments were fixed in 4% paraformaldehyde, automatically processed, paraffin-embedded and mounted onto slides. Blank slides were used to posterior staining methods and measurements as described ahead:

***Picrossirius Red*** stain was used to measure interstitial collagen. Ten random images of 200X magnification of each, the border and remote area of MI were quantified per animal. The values are the percentage of positive *Picrossirius red* fibers per total area of the image. The final values to be compared are the mean value of the animals in each experimental group. Image recording and automatic measurements were performed using the software Leica QWin 3 (Leica QWin Plus V 3.5.1 –Leica Microsystems).

***Periodic acid-Schiff*** stain was used to quantify the total number of vessels in LV areas. Fifteen random images of 400X magnification of the MI, border and remote areas of LV were quantified per animal. The values shown represent the mean of the vessel number obtained in all the images per area in each experimental group. Vessel quantification was performed manually by two trained operators who were blinded to the experimental groups.

***Polarized light microscopy*** assessments to measure “scar maturity” were performed as described by Whittaker and colleagues [53]. Twenty random images of 400X magnification were recorded from *Picrossirius red* stained slides from MI area by polarizer filter coupled light microscope connected to an image scanning system (Leica Imaging Systems). Percentage of red (mature and little flexible), yellow (intermediate) and green (immature, and more flexible) fiber was automatically calculated using the software Leica QWin 3 (Leica QWin Plus V 3.5.1). Data were shown as the percentage of each fiber (by color) per total of fibers comparing placebo and 4 million pASC/kg groups. Non-additional staining or immunoreactions were performed in these material. Image recording and quantification was performed by trained operator who was blinded to the experimental groups (more details in Supporting material).

***Hematoxylin & Eosin*** stain was performed, and cellular immune rejection of the injected cells were assessed according to *Malliaras et al* [54]. Twenty random images of 200X magnification of MI, border and remote areas of LV were recorded. Inflammatory infiltrates were qualified and quantified by a simplified method based on the *ISHLT consensus report* [55,56]. All the measurements were performed by two trained operators who were blinded to the experimental groups.

#### Immunohistochemistry and quantification

Immunohistochemical reactions were performed according to the protocol described below to assess and quantify the type of collagen present in the scar and the type of inflammatory cells observed in the LV areas after 30 days of cell injection. Briefly, after tissue processing described above, blank slides were subjected to deparaffinization followed by antigenic recovery by high-temperature sodium citrate reaction to expose the epitopes. Endogenous peroxidase activity was blocked using a 3% solution of hydrogen peroxide (H_2_O_2_). The sections were blocked with 2% BSA solution and then incubated overnight primary antibodies (details in Table A in S1 File). Next, the slides were incubated with anti-IgG secondary antibody conjugated with biotin followed by 30 minutes of incubation with streptavidin and stained with diaminobenzidine (DAB—Zymed).

***The quantification of the percentage of Collagen types I and III*** was performed in slides obtained from papillary level tissues of MI area to compare placebo and 4 million pASC/kg groups. Immunohistochemical staining were performed using specific antibodies. Twenty random images of 200X magnification were recorded and following measured using Leica QWin 3 software (Leica QWin Plus V 3.5.1). Data were presented as the percentage of positive stain per area of tissue. All the measurements were performed by a trained operator who was blinded to the experimental groups.

***The quantification of the percentage of CD3 and CD45RA inflammatory cells*** was performed in MI, border and remote areas to compare the four experimental groups. Immunohistochemical staining were performed using specific antibodies. Twenty random images of 200X magnification were recorded and following measured using Leica QWin 3 software (Leica QWin Plus V 3.5.1). Data were presented as the percentage of positive stain per area of tissue. All the measurements were performed by a trained operator who was blinded to the experimental groups.

### DNA and RNA extractions and PCR

Genomic DNA was extracted from the LV border of MI area of frozen tissues using the Proteinase K lysis/isopropanol precipitation protocol. PCRs were performed according to a previous protocol [57], using Taq platinum-DNA-polymerase kit (Invitrogen) and AMEL primer pairs (Table B in S1 File). PCR-products were applied onto a 2% agarose gel.

Total RNA was extracted from frozen tissues of LV areas (MI, border and free wall) by the single-step method using Trizol reagent (Invitrogen, Carlsbad, CA) according to the manufacturer’s instructions. cDNA synthesis from total RNA (5 μg) was produced by reverse transcription (RT) using the superscript III kit according to the manufacturer’s protocol (Invitrogen). Polymerase chain reaction (PCR) was performed to test the primers (Table B in S1 File) using pig fibroblasts and endothelial cells of mammary artery cDNAs as a template, and the reactions were performed using Taq-polymerase manufacturing protocol (Invitrogen) (more details in Supporting material).

### Statistical analysis

Results were expressed as the mean ± standard error of the mean (SEM). One- or two-way analysis of variance (ANOVA) with Bonferroni *posthoc test* or unpaired Student’s t-test were utilized to compare groups as appropriate. All statistical analyses were performed using GraphPad Prism 7.0 (GraphPad Software Inc., CA, USA). All data were shown as means ± SEM. P-values < 0.05 were considered significant. For p<0.05 = *; p<0.005 = **; p<0.0005 = *** and p<0.0001 = ****.

## Supporting information

S1 File A PDF file containing: Supplemental Figures, Tables and material and methods details. (DOCX)
