# Supplementary material for: Allogeneic pASC transplantation in humanized pigs attenuates cardiac remodeling post-myocardial infarction
Source: PLoS One. 2017 Apr 27;12(4):e0176412. doi: 10.1371/journal.pone.0176412 (PMC5407644; doi:10.1371/journal.pone.0176412)
Supplement: S1 File — (DOCX) [file pone.0176412.s001.docx]

**Allogeneic pASC transplantation in humanized pigs attenuates cardiac remodeling post-myocardial infarction**

**Supporting results and material and methods**

Rafael Dariolli, Marcus V Naghetini, Euclydes F Marques, Celso K Takimura, Leonardo S Jensen, Bianca Kiers, Jeane M Tsutsui, Wilson Mathias Jr., Pedro A Lemos Neto, and Jose E Krieger*.

*Heart Institute (InCor), University of São Paulo Medical School, São Paulo, Brazil*

***Please address correspondence and request for reprints to:** José E. Krieger, M.D., Ph.D., Lab. of Genetics and Molecular Cardiology/LIM 13, Heart Institute (InCor), University of São Paulo Medical School, Av. Dr. Enéas C. Aguiar 44, 05403-000 São Paulo, SP, Brazil.

Telephone: 55 11 2661 5068; fax: 55 11 2661 5022; e-mail: [krieger@incor.usp.br](mailto:krieger@incor.usp.br), <http://www.incor.usp.br/genetica>

**Keywords:** Perfusion, myocardial infarction, stem cells, pigs, collagen

***Supplementary material***

**S1 Figure**: **Experimental design of cell therapy protocol and details of cell injection, biological material processing, and macroscopical measurements**. A) To test the additive effects of cell therapy in humanized pigs, they first were subjected to a basal angiography and then to a catheter-based method to induce MI (phase I). After 24 hours of LCx occlusion, all the pigs received a daily drug treatment (iACE and beta-blocker). Thirty days after occlusion, in phase II, pigs were subjected to basal echocardiographic examinations and then to surgical intramyocardial injection of pASC in different doses. Finally, in phase III, thirty days after cell injection (60 days after occlusion) pigs were subjected to final examinations (angiography and echocardiography) and killed to other assessments. B) Number of animals per group. C) Schematic representation of the targeted areas injected with stem cells. D) After killed, the heart of pigs were collected and dissected to obtain only left ventricle (LV). LV was sliced in 7 parts (5mm approx. base to apex). E) To measure wall thickness an average of 3 different measures was performed as indicated by green and yellow lines respectively in remote and MI areas. These measurements were performed in macroscopic slices of LV in papillary level, excluding papillary muscles and another accessory musculature.

**
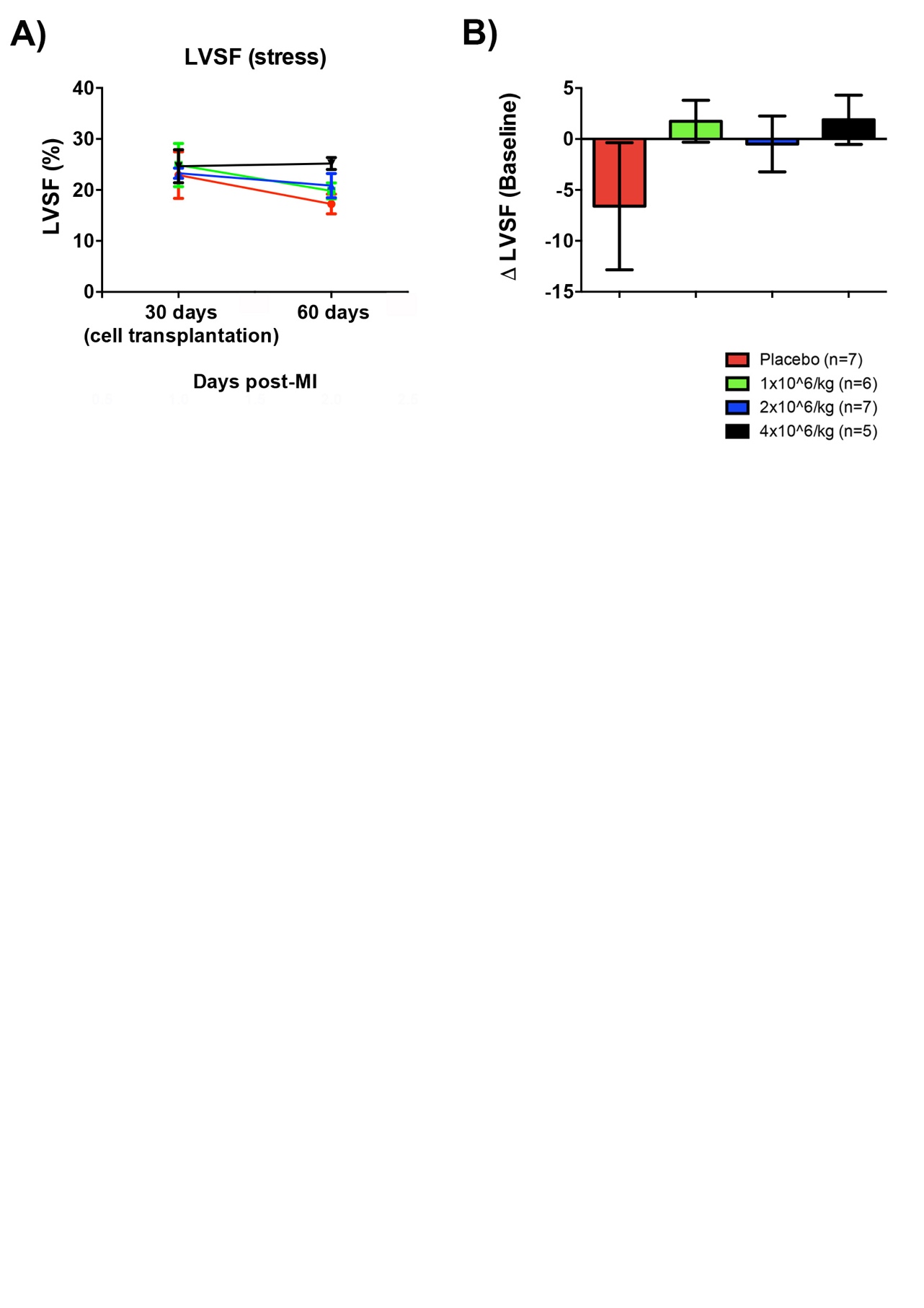
**

**Figure B: Echocardiographic cardiac functional parameters**. A) LVSF are shown for each group, before injection of pASC (30 days after MI induction) and 60 days after injection (before euthanasia). B) Comparison of the absolute changes in LVSF. ΔLVSF are shown a trend to reduction of deterioration of shortening capacity toward the stem cells therapies, specially in highest amount of cells injected in LV. All data are means ± SEM. P > 0.05 in all the comparisons.


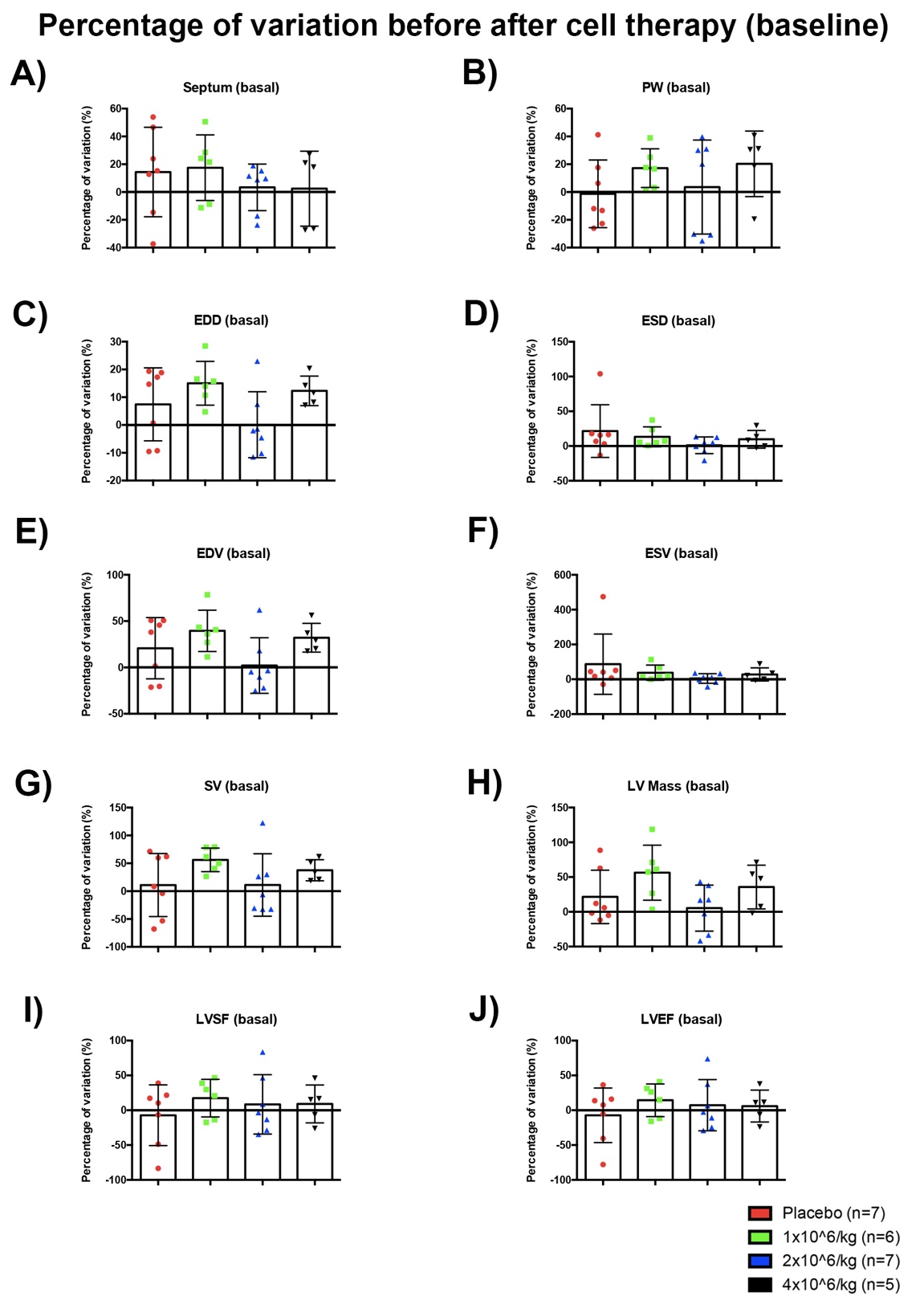


**Figure C: Echocardiographic assessments: percentage of variation of cardiac morphological and functional parameters in the basal state (before pharmacological stress using dipyridamole)**. The percentage of variation was calculated by post-therapy and pre-therapy parameters ([post-therapy value – pre-therapy value / pre-therapy value] x100. A) Percentage of variation of septum size. B) Percentage of variation of posterior wall size (PW). C) Percentage of variation of end diastolic diameter (EDD). D) Percentage of variation offend systolic diameter (ESD). E) Percentage of variation offend diastolic volume (EDV). F) Percentage of variation of end systolic volume (ESV). G) Percentage of variation of stroke volume (SV). H) Percentage of variation of left ventricle mass (LV mass). I) Percentage of variation of left ventricle shortening fraction (LVSF). J) Percentage of variation of left ventricle ejection fraction (LVEF). All data are means ± SEM. P > 0.05 in all the comparisons.

**
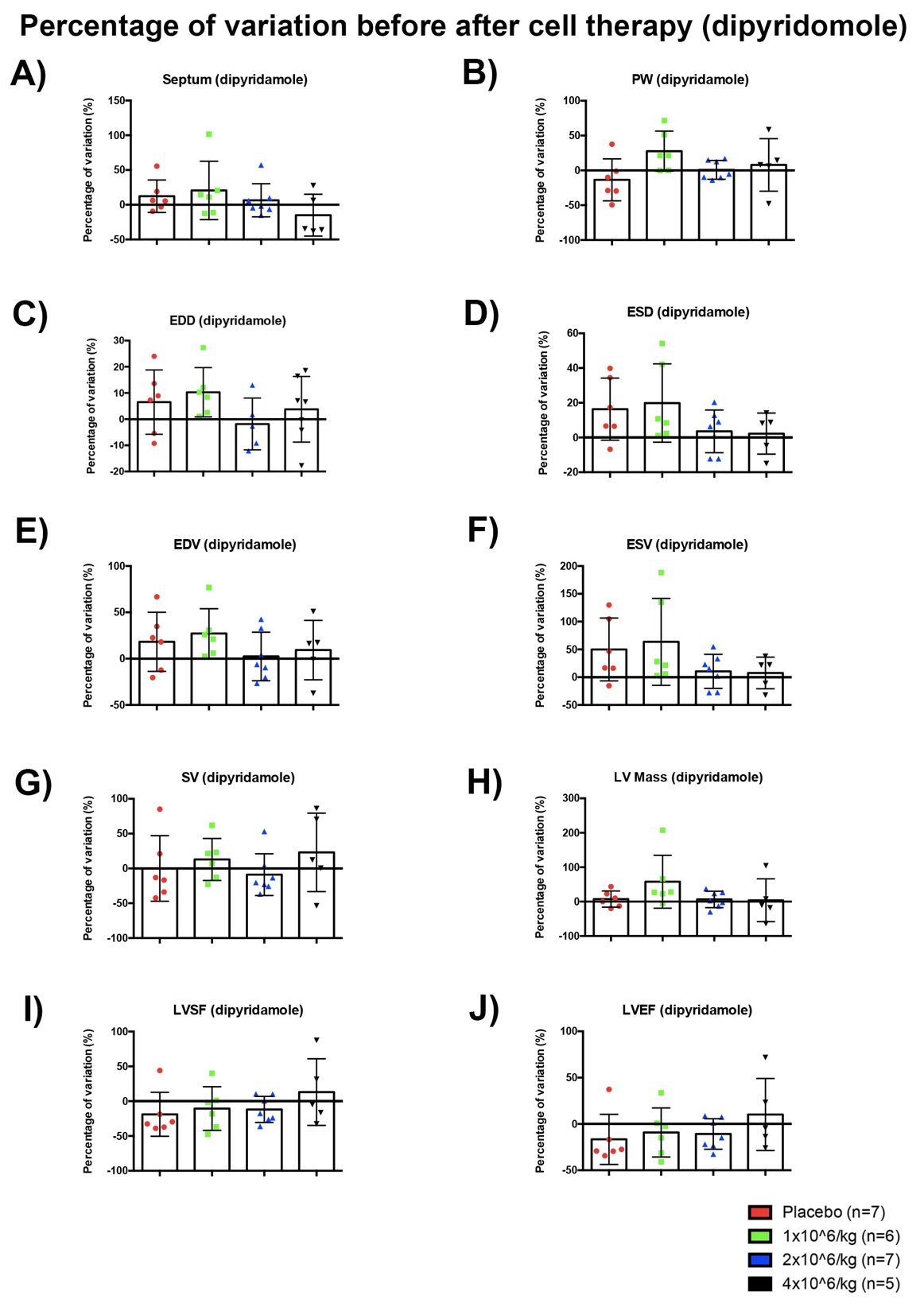
**

**Figure D: Echocardiographic assessments: percentage of variation of cardiac morphological and functional parameters in the stimulated state (after pharmacological stress using dipyridamole)**. The percentage of variation was calculated by post-therapy and pre-therapy parameters ([post-therapy value – pre-therapy value / pre-therapy value] x100. A) Percentage of variation of septum size. B) Percentage of variation of posterior wall size (PW). C) Percentage of variation of end diastolic diameter (EDD). D) Percentage of variation offend systolic diameter (ESD). E) Percentage of variation offend diastolic volume (EDV). F) Percentage of variation of end systolic volume (ESV). G) Percentage of variation of stroke volume (SV). H) Percentage of variation of left ventricle mass (LV mass). I) Percentage of variation of left ventricle shortening fraction (LVSF). J) Percentage of variation of left ventricle ejection fraction (LVEF). All data are means ± SEM. P > 0.05 in all the comparisons.

**
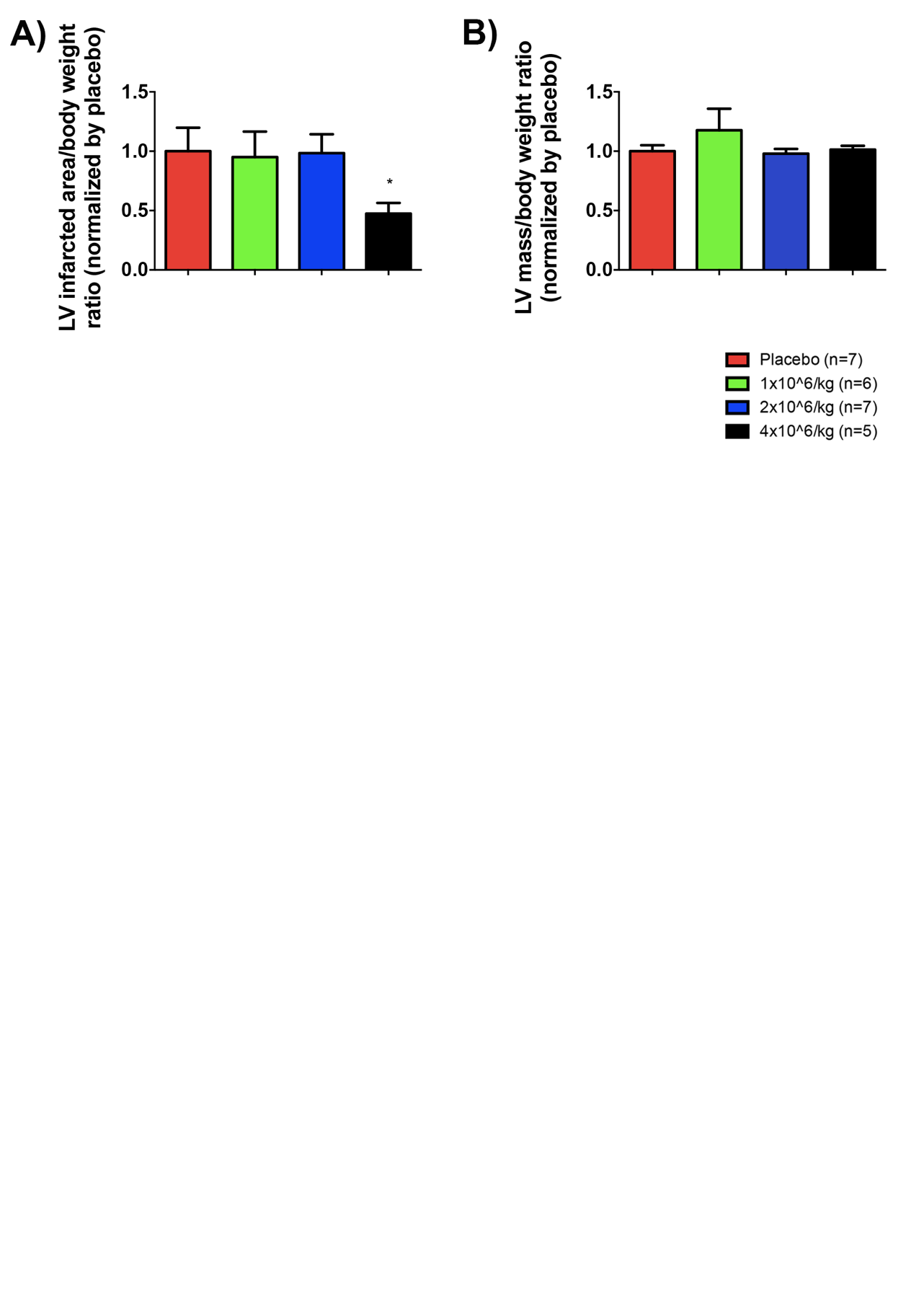
**

**Figure E: Four million/Kg of allogeneic pASC reduces LV injured area**. A) Ratio between LV infarcted area and body weight normalized by placebo group. B) Ratio between LV mass and body weight normalized by placebo group. No differential hypertrophy was observed between groups assessing LV mass per body weight mass.


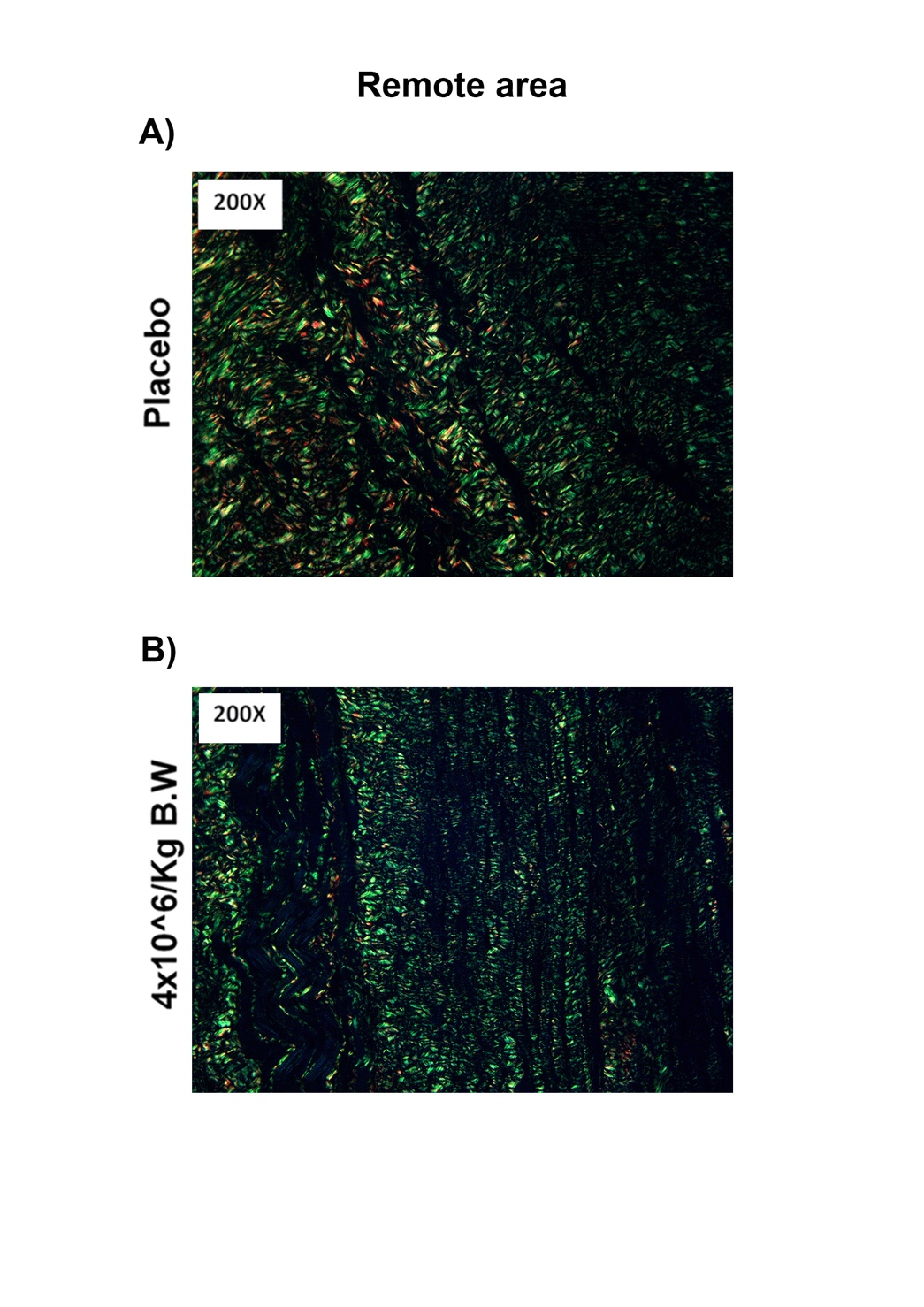


**Figure F: Polarized light microscopy – Collagen fiber maturity**. A) Representative image of placebo LV section from remote area. B) Representative image of 4 million cell/Kg LV section from remote area. Note that there were not qualitatively strong evidences of differences in the remote tissue comparing highest dose of cell group vs. placebo.

**Table A:** Antibodies list and details.

**Table B:** Primer sequences, primer melting temperature and expected product lengths.

**
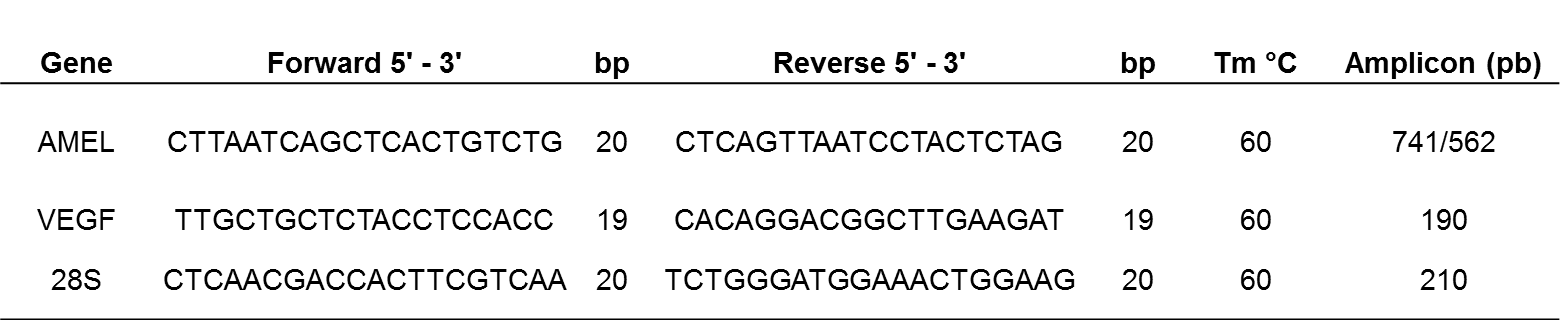
**

| After Stress |  | Placebo (n=7) | | | | | | 1x10^6^ pASC/Kg (n=6) | | | | | | 2x10^6^ pASC/Kg (n=7) | | | | | | 4x10^6^ pASC/Kg (n=5) | | | | | |
| --- | --- | --- | --- | --- | --- | --- | --- | --- | --- | --- | --- | --- | --- | --- | --- | --- | --- | --- | --- | --- | --- | --- | --- | --- | --- |
|  |  | Before injection | | | After injection | | | Before injection | | | After injection | | | Before injection | | | After injection | | | Before injection | | | After injection | | |
| Parameters |  | Mean | ± | SEM | Mean | ± | SEM | Mean | ± | SEM | Mean | ± | SEM | Mean | ± | SEM | Mean | ± | SEM | Mean | ± | SEM | Mean | ± | SEM |
| MI area | (%) | 10.78 | ± | 2.97 | 10.07 | ± | 3.45 | 9.82 | ± | 2.34 | 9.81 | ± | 2.51 | 13.03 | ± | 1.70 | 10.51 | ± | 3.75 | 11.65 | ± | 0.71 | 8.13 | ± | 1.92 |
| Septum | (mm) | 0.55 | ± | 0.12 | 0.61 | ± | 0.13 | 0.54 | ± | 0.07 | 0.62 | ± | 0.10 | 0.52 | ± | 0.03 | 0.54 | ± | 0.08 | 0.57 | ± | 0.13 | 0.56 | ± | 0.10 |
| PW | (mm) | 0.44 | ± | 0.08 | 0.42 | ± | 0.06 | 0.39 | ± | 0.03 | 0.45 | ± | 0.05 | 0.42 | ± | 0.08 | 0.42 | ± | 0.10 | 0.43 | ± | 0.08 | 0.50 | ± | 0.02 |
| EDD | (mm) | 4.14 | ± | 0.35 | 4.44 | ± | 0.55 | 4.30 | ± | 0.24 | 4.95 | ± | 0.51 | 4.56 | ± | 0.54 | 4.55 | ± | 0.64 | 3.99 | ± | 0.45 | 4.47 | ± | 0.36 |
| ESD | (mm) | 3.11 | ± | 0.68 | 3.60 | ± | 0.52 | 3.40 | ± | 0.33 | 3.83 | ± | 0.36 | 3.60 | ± | 0.50 | 3.62 | ± | 0.52 | 3.06 | ± | 0.39 | 3.33 | ± | 0.35 |
| LVSF | (%) | 25.49 | ± | 12.26 | 18.89 | ± | 6.52 | 20.69 | ± | 8.51 | 22.44 | ± | 6.31 | 21.11 | ± | 6.24 | 20.63 | ± | 2.10 | 23.25 | ± | 5.01 | 25.14 | ± | 7.17 |
| EDV | (cm^3^) | 76.87 | ± | 15.07 | 91.63 | ± | 27.00 | 83.39 | ± | 11.14 | 117.38 | ± | 29.69 | 97.34 | ± | 25.88 | 97.78 | ± | 30.58 | 71.09 | ± | 17.78 | 91.68 | ± | 16.93 |
| ESV | (cm^3^) | 41.20 | ± | 20.40 | 56.16 | ± | 19.70 | 48.14 | ± | 11.11 | 63.81 | ± | 15.26 | 55.98 | ± | 17.24 | 56.79 | ± | 17.96 | 37.85 | ± | 11.54 | 46.05 | ± | 12.31 |
| SV | (cm^3^) | 35.67 | ± | 11.17 | 35.47 | ± | 15.35 | 35.25 | ± | 14.90 | 53.57 | ± | 21.09 | 41.36 | ± | 15.03 | 40.99 | ± | 13.51 | 33.24 | ± | 10.93 | 45.63 | ± | 14.73 |
| LVEF | (%) | 48.55 | ± | 18.90 | 38.60 | ± | 11.75 | 41.41 | ± | 14.94 | 44.47 | ± | 10.61 | 42.50 | ± | 10.87 | 42.17 | ± | 3.81 | 46.77 | ± | 8.30 | 49.12 | ± | 11.25 |
| LV Penn Mass | (g) | 53.98 | ± | 8.82 | 64.32 | ± | 18.87 | 53.22 | ± | 8.24 | 84.20 | ± | 28.80 | 60.64 | ± | 11.92 | 64.08 | ± | 21.72 | 51.54 | ± | 13.06 | 67.93 | ± | 15.74 |

**Table C:** Baseline echocardiographic linear measurements to assess cardiac function before and after cell injection. Data were represented by means ± SEM. P>0.05.

MI: myocardial infarction, PW: posterior wall, EDD: end-diastolic diameter, ESD: end-systolic diameter, LVSF: left ventricle shortening fraction, EDV: end-diastolic volume, ESV: end-systolic volume, SV: stroke volume, LVEF: left ventricle ejection fraction, LV: left ventricle.

**Table D:** Homemade grading system for rejection at 8 weeks post-MI and 4 weeks post cell transplantation (frequency of Inflammatory infiltrated observation/total observation per area).


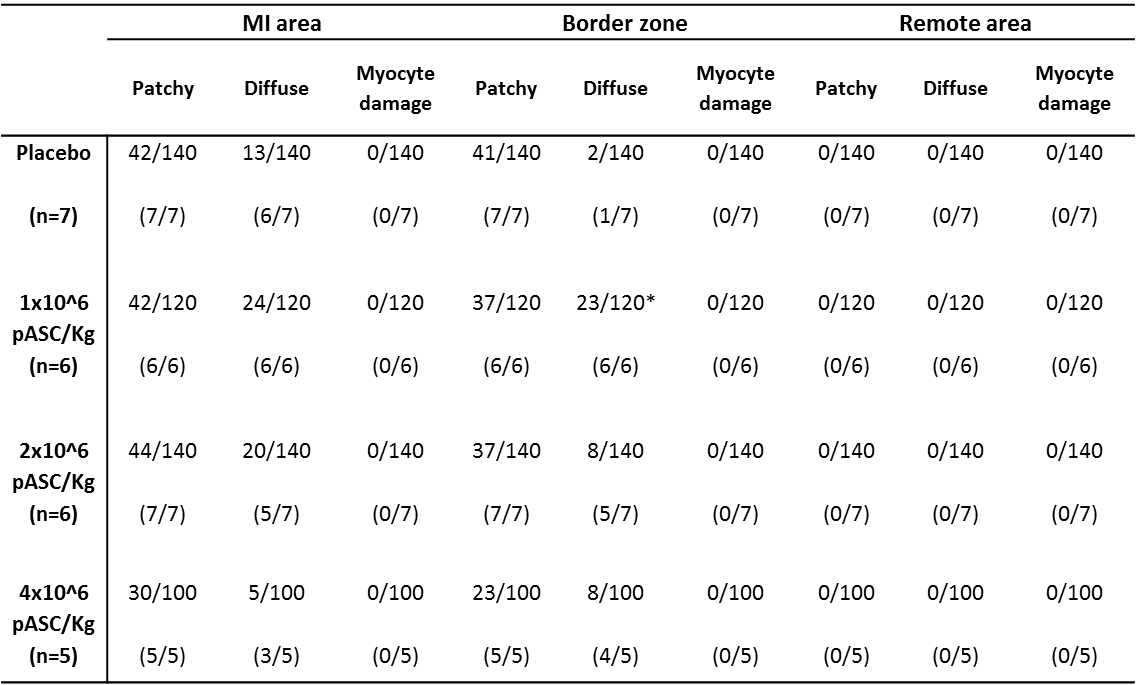


***Supplementary material and methods***

***Animal procedures***

Pigs were maintained in a local commercial swine farm (Granja RG, Suzano-SP, Brazil) with free access to food and water during the protocol. The animals were acclimated at least per 24h before induction of coronary occlusion procedure. All pigs were fasted overnight before coronary occlusion and before intramyocardial injection procedures. The animals were sedated with a mixture of ketamine chloridrate (8 mg/kg, Vetbrands) and midazolam hydrochloride (0.5 mg/kg; Roche). After 10 to 20 min, a cannula was introduced into a superficial ear vein. The anesthesia was induced with sodium thiopental (12.5 mg/ kg; Cristalia) and then orotracheal intubation (7- to 7.5-mm tube - Chilecom) was performed. Anesthesia was maintained with isoflurane (1.5% to 2.5% - Baxter - in 100% oxygen – in anesthetic equipment (Origami Ergo System - Takaoka). Before the procedure, the pigs received intramuscular benzathine penicillin 1.2 million units (Eurofarma) to avoid infections. After the procedure, the pigs were treated with intramuscular sodium dipyrone (1000mg, 4 times; 2 doses daily - Sanofi Aventis) to minimize pain. To prevent episodes of ventricular fibrillation, 2% lidocaine hydrochloride (1 mg/kg/hour venous infusion - Cristalia) was administrated intravenously in physiological saline solution during the coronary occlusion and during surgical procedures of cell injection. When the ventricular fibrillations were unavoidable, electrical cardioversion was performed (Codemaster XL - Hewlett Packard) by applying 200-300 J with the paddles pressed to the anterior chest wall along with close-chest cardiac massage. Cardioversion attempts were extended at least for 30 min after fibrillation start. The fibrillation was considered irreversible after this period. In the end of the protocol the pigs were killed with a high dose of sodium thiopental (25-50 mg/kg; Cristalia) followed by an overdose of potassium chloride (KCl 19.1% solution 1mL/Kg).

***Epicardial electrical mapping***

In order to create a homogeneous protocol of cell injections, we designed a simple and reproducible strategy to help to identify the border zone of the MI in pigs during the surgical procedures of intramyocardial injection of pASCs. Based on the ECG signal we performed a epicardial electrical mapping of the LV using external electrodes plugged in the regular spots over the skin and a electrode plugged in a metal edge able to be manipulated in direct contact with LV epicardial muscle. Electrical mapping real-time information associated with Wall Motility (WM) information obtained before by echocardiography and also visual analysis of LV injured areas improved significantly our abilities to inject cell aliquots in the relevant border areas. Briefly, animals were monitored using a 5 electrode ECG monitor. The left superior electrode was unplugged from the skin and plugged in a disconnected of the system*“electric bistoury pen”* directly in the metal plug (Fig1A). The gain of the ECG monitor was reduced to N/4 to improve signal quality (Fig.1B). The metallic edge of the electric bistoury pen was used as the fifth electrode. The contact of the metallic edge with the heart wall was able to close the circuit generating electrical recording in the ECG monitor (Fig1B and C). The contact of bistoury with the remote area as able to generate a relatively normal ECG signal. This signal was considered as the representative of a remote and electrically competent area. Moving the metallic edge of bistoury towards the injured zone, a gradual change in the electrical signal pattern could be recorded. The absence of electrical signal was observed in the middle of the scars. Based on the pattern of electrical signal in the wall of LV we could properly visualize the limits of the MI and border areas and so, perform the cell injections with high precision.


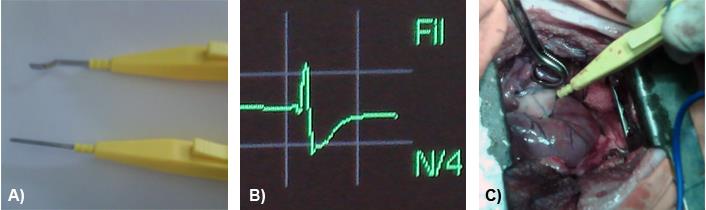


**Figure 1: *Epicardial electrical mapping*.** A) electric bistoury pen. Note that we modify curvature of the metallic edges in order to not damage the injured wall. B) ECG monitor gain in N/4 and electrical signal from a border of MI area. C) Epicardial electrical mapping of LV during procedures of cell injection. All the material used to perform electrical mapping was manipulated in sterile conditions.

***Echocardiography***

Echocardiographic imaging was adjusted to optimize image acquisition, and echocardiographic parameters were then held constant for each experiment. Animals were positioned in the left lateral decubitus for imaging acquisition. Commercially available ultrasound systems (Sonos 7500 - Philips Medical Systems, Bothell, Washington, USA) were used to acquire the images. Linear measurements, such as left ventricular end-diastolic and end-systolic diameters, were obtained in parasternal long-axis images. Fractional shortening was determined as the difference of end-diastolic and end-systolic diameters divided by the end-diastolic diameter. Left ventricular volumes and ejection fraction were determined using the *Teichholz* formula with left ventricular dimensions. The end-diastolic and end-systolic areas of LV were measured by planimetry in parasternal-papillary short-axis images.

In the last years, real-time myocardial perfusion echocardiography (RTMPE) has been demonstrated a useful technique for simultaneous evaluation of wall motion and myocardial perfusion (MP) [1,2]. Quantitative RTMPE has the potential advantage to be less dependent on observer experience and allows for determining myocardial blood flow and reserve [3–7]. This technique, although time-consuming was shown useful for evaluating different clinical conditions with alteration in microvascular blood flow [8,9].

**RTMPE**

The protocol was in accordance with the position of the American Heart Association Guidelines for Animal Research Use [10]. Echocardiographic imaging was adjusted to optimize image acquisition and echocardiographic parameters were then held constant for each experiment. Animals were positioned in the left lateral decubitus for imaging acquisition. Commercially available ultrasound systems (Sonos 7500 - Philips Medical Systems, Bothell, Washington, USA) equipped with an S3 broadband transducer and contrast echo software with low-mechanical index imaging was used for RTMPE. RTMPE imaging was performed in the parasternal long-axis, parasternal short-axis, apical 2-chamber, and apical 4-chamber views using power modulation mode with a mechanical index of 0.2, frame rate 25-30 Hz, and continuous intravenous infusion of lipid-encapsulated microbubbles Definity® (Lantheus Medical Imaging, Inc., N. Billerica, Massachusetts, USA) or Sonovue® (Bracco, Milan, Italy). Microbubble destruction was achieved using a packet of five high-intensity (mechanical index 1.5) pulses (flash). Ultrasound contrast agent Definity® or Sonovue® was diluted in 60 mL saline solution and infused continuously into the ear vein after an initial bolus dose of 3 mL. Contrast infusion rate was adjusted till complete left ventricular cavity opacification and shadowing in the mid left atrial cavity was obtained. Once a stable myocardial enhancement was reached, the contrast infusion rate was kept constant, and sequences of low-power perfusion images containing at least 15 cardiac cycles were acquired. After completion of resting perfusion sequences, contrast infusion was stopped, and hyperemia was induced by dipyridamole infusion via a parallel port. A total dose of 0.56 mg/Kg of dipyridamole was infused during 4 minutes. In the end of dipyridamole infusion, the second acquisition of images was performed following the same settings as a baseline.

**Qualitative Analysis of Wall Motility (WM) and Myocardial Perfusion (MP)**

Qualitative analysis of segmental myocardial contraction was based on visual assessment of myocardial thickening during optimal cavity contrast enhancement and was graded according to a scoring system as follows: 1-normal, 2-hypokinesis, 3-akinesis and 4-dyskinesis. Qualitative analysis of MP was based on a visual assessment of subendocardial and transmural myocardial contrast enhancement after the flash impulse. The myocardial opacification during this phase was graded visually as follows: grade 1, intense; grade 2, reduced; grade 3, lack of opacification in the first 4 to 5 cardiac cycles following the flash impulse. The reduced myocardial opacification (grade 2) could represent either reduced intensity at all times and/or delayed appearance of contrast either compared to other segments or requir >2 s during stress [11].

**Quantitative Analysis of RTMPE**

Off-line image analysis for quantification was performed with commercially available software (Q-lab 6.0 Phillips Medical Systems, Bothell, WA, USA). Myocardial blood flow was quantified in the infarcted areas, normal areas and in the borders zone. Regions of interest were traced manually within the selected segments in the myocardium and in the adjacent left ventricular cavity. Myocardial plateau (A_M_, dB) and adjacent left ventricular plateau (A_LV_, dB) signal intensities, and signal intensity exchange rate (ß, s^-1^) – that is proportional to myocardial blood flow velocity - were calculated automatically by the software. Normalized myocardial acoustic intensity (An) was defined as the ratio of A_M_ divided by A_LV_, representing the relative myocardial blood volume [3,6,12]. To originate an index of blood flow, the product of An and ß (s-1) was calculated. The intra-observer variability for the measurements of ß reserve and MBF in our laboratory have been previously reported as 2.1% (r=0.99) and 7.4% (r=0.95), respectively. The inter-observer variability for the same parameters were 6.8% (r=0.98) and 5.5% (r=0.97), respectively [4,5].

***Anatomopathological Analyses***

After terminal functional assessment, the animals that were still under anesthesia were sacrificed by an overdose of potassium hydrochloride (KCl). After cardiac arrest, the heart was removed and dissected to obtain the left ventricle (LV) as previously described by *Dariolli et al*. [13]. Briefly, LV was sectioned transversely from the base to apex in 5-mm sections (on average 7 sections, Fig. S1D) and processed with specific stain techniques.

- 1. **Macroscopic assessment**

LV sections 1 and 4-7 were stained with 2,3,5 Triphenyl tetrazolium chloride (TTC) for macroscopical assessments. Briefly, a solution of 1% TTC in phosphate buffer pH 7.4 was used. The cross-sections were incubated in TTC at 37 °C for 15-20 minutes and then incubated with 10% formaldehyde. Boards were mounted with all the slices and images. ***The area of ​​necrotic tissue*** was measured by planimetry. The ***thinning ratio*** was determined as the ratio of averages of remote wall thickness and injured wall thickness. Wall thicknesses were obtained from the middle section (section 4) and measured in triplicate (Fig. S1E). All the measurements were obtained using ImageJ software [14].

- 1. **Microscopic assessment**

LV section number 3 was divided into three segments: remote, border zone and injured area. These segments were fixed in 4% paraformaldehyde. Forty-eight hours after fixation, the tissues were placed in plastic cassettes and processed in a total cycle of 12 hours for dehydration, diaphanization, and paraffinization (Leica TP1020 - Leica). The paraffin-embedded tissues were sectioned on a microtome (4 µm thick) and mounted onto slides and subjected to different staining protocols. ***Picrossirius Red*** stain was used to measure interstitial collagen. Ten random images with 200X magnification of the injured and remote area of MI were quantified per animal. The values showed the mean of the animals in each experimental group. Images of the slices were obtained using a software system of image acquisition and the measurements by Leica QWin 3 (Leica QWin Plus V 3.5.1 – Leica Microsystems). The ***Periodic acid-Schiff*** stain was used to quantify the number of vessels in LV areas. Fifteen random images with 400X magnification of the injured, border and remote area of LV were quantified per animal. The values shown represent the mean of the vessel number obtained in all the images per area of interest in each experimental group. Vessel quantification was performed manually by two trained operators who were blinded to the experimental groups. ***Hematoxylin & Eosin*** stain was performed, and cellular immune rejection of the injected cells were assessed according to *Malliaras et al* [15]. Briefly, twenty random 200X magnification images of MI, border zone and remote area of LV were evaluated by two blinded trained operators. Inflammatory infiltrates were qualified and quantified based on the *ISHLT consensus report* [16] for rejection.

***Polarized light microscopy***

To assess “scar maturity” we performed polarized light microscopy technics as previous described by Whittaker and colleagues [17]. Twenty random images of 400X magnification were recorded from *Picrossirius red* stained slides from MI area by polarizer filter coupled light microscope connected to an image scanning system (Leica Imaging Systems). Percentage of red (mature and little flexible), yellow (intermediate) and green (immature, and more flexible) fiber was automatically calculated using the software Leica QWin 3 (Leica QWin Plus V 3.5.1). Previous to the image recording the *“image recording and quantification”* software was configured to take and quantify images in HSI (Hue, Saturation and Intensity) format. To the red acquisitions HSI was: H: 16, 0; S: 255, 151 e I: 232, 12. To the yellow acquisitions HSI was: H: 34, 0; S: 210, 109; I: 253, 55. To the green acquisitions HSI was: H: 126, 13; S: 255,109 e I: 99, 9. All the images were acquired using those configuration for the samples. Data were shown as the percentage of each fiber (by color) per total of fibers comparing placebo and 4 million pASC/kg groups. Non-additional staining or immunoreactions were performed in these material. Image recording and quantification was performed by trained operator who was blinded to the experimental groups

***Immunohistochemistry***

Immunohistochemical reactions were performed according to the protocol described below to assess and quantify the type of collagen present in the scar and the type of inflammatory cells observed in the LV areas after 30 days of cell injection. The slides were obtained from papillary level tissues of MI area to collagen assessments and from MI, border and free wall areas to inflammatory assessments. Briefly, after tissue processing, histological slides were obtained and subjected to deparaffinization. The slides were then subjected to antigenic recovery by high-temperature sodium citrate reaction to expose the epitopes. Endogenous peroxidase activity was blocked using a 3% solution of hydrogen peroxide (H_2_O_2_) for 10 minutes. The sections were blocked with 2% BSA solution for one hour at room temperature and then incubated overnight at 4°C with primary antibodies (details in Table S1). Next, the slides were washed and incubated for 1 hour at room temperature with anti-IgG secondary antibody conjugated with biotin followed by 30 minutes of incubation with streptavidin and then a 5-minute stain with diaminobenzidine (DAB – Zymed). A counterstain with hematoxylin was performed. Quantification of different types of collagen and inflammatory cells was performed by analysis of the stained areas using Leica software QWin 3 (Leica QWin Plus V 3.5.1 – Leica Microsystems).

***DNA extraction and PCR***

Genomic DNA was extracted from the frozen tissues of LV border area of MI using the isopropanol precipitation protocol. Briefly, 0.5 mg of tissue were incubated in lyses buffer (100 mM Tris-NaCl, 5 mM EDTA, 0,2% SDS, 200 mM NaCl and 20 μg/μl of Proteinase K) at 56°C for 60 minutes. After lysis, isopropanol was added to the samples to precipitate DNA. According to a previous modified protocol [18], 40 ng of extracted DNA was added to a 10-μl PCR reaction mixture containing 0.4 μM of AMEL-F and AMEL-R primer (S2 Table), 0.4 mM of each dNTP, 3 mM MgCl_2_, 1 unit of Taq platinum-DNA-polymerase (Invitrogen) and 1x PCR-buffer. The reaction conditions were the following: initial denaturation for 3 min at 95 °C followed by 35 cycles of denaturation for 30 s at 95 °C, primer annealing for 30 s at 60 °C and elongation for 45 s at 72 °C. The final elongation step was performed for 5 min at 72 °C. PCR-products (XY 741 bp and 562 bp and XX only single 741 bp band) were applied onto a 2% agarose gel.

***RNA extraction and RT-PCR***

Total RNA was extracted from frozen tissues of LV areas (MI, border and free wall) by the single-step method using Trizol reagent (Invitrogen, Carlsbad, CA) according to the manufacturer’s instructions. cDNA synthesis from total RNA (5 μg) was produced by reverse transcription (RT) using the superscript III kit according to the manufacturer’s protocol (Invitrogen). Polymerase chain reaction (PCR) was performed to test the primers (S2 Table) using pig fibroblasts and endothelial cells of mammary artery cDNAs as a template, and the reactions were performed using Taq-polymerase manufacturing protocol (Invitrogen).

**References**

1. Porter TR, Xie F, Silver M, Kricsfeld D, Oleary E. Real-time perfusion imaging with low mechanical index pulse inversion Doppler imaging. J Am Coll Cardiol. 2001;37: 748–53. Available: http://www.ncbi.nlm.nih.gov/pubmed/11693747

2. Shimoni S, Zoghbi WA, Xie F, Kricsfeld D, Iskander S, Gobar L, et al. Real-time assessment of myocardial perfusion and wall motion during bicycle and treadmill exercise echocardiography: comparison with single photon emission computed tomography. J Am Coll Cardiol. 2001;37: 741–7. Available: http://www.ncbi.nlm.nih.gov/pubmed/11693746

3. Wei K, Jayaweera AR, Firoozan S, Linka A, Skyba DM, Kaul S. Quantification of myocardial blood flow with ultrasound-induced destruction of microbubbles administered as a constant venous infusion. Circulation. 1998;97: 473–83. Available: http://www.ncbi.nlm.nih.gov/pubmed/9490243

4. Osório AFF, Tsutsui JM, Kowatsch I, Guerra VC, Ramires JAF, Lemos PA, et al. Evaluation of blood flow reserve in left anterior descending coronary artery territory by quantitative myocardial contrast and Doppler echocardiography. J Am Soc Echocardiogr. 2007;20: 709–16. doi:10.1016/j.echo.2006.11.016

5. Kowatsch I, Tsutsui JM, Osório AFF, Uchida AH, Machiori GGA, Lopes ML, et al. Head-to-head comparison of dobutamine and adenosine stress real-time myocardial perfusion echocardiography for the detection of coronary artery disease. J Am Soc Echocardiogr. 2007;20: 1109–17. doi:10.1016/j.echo.2007.02.008

6. Vogel R, Indermühle A, Reinhardt J, Meier P, Siegrist PT, Namdar M, et al. The quantification of absolute myocardial perfusion in humans by contrast echocardiography: algorithm and validation. J Am Coll Cardiol. 2005;45: 754–62. doi:10.1016/j.jacc.2004.11.044

7. Reant P, Labrousse L, Lafitte S, Tariosse L, Bonoron-Adele S, Padois P, et al. Quantitative analysis of function and perfusion during dobutamine stress in the detection of coronary stenoses: two-dimensional strain and contrast echocardiography investigations. J Am Soc Echocardiogr. 2010;23: 95–103. doi:10.1016/j.echo.2009.10.001

8. Lima MF, Mathias W, Sbano JCN, de la Cruz VY, Abduch MC, Lima MSM, et al. Prognostic value of coronary and microvascular flow reserve in patients with nonischemic dilated cardiomyopathy. J Am Soc Echocardiogr. 2013;26: 278–87. doi:10.1016/j.echo.2012.12.009

9. Santos JMT, Kowatsch I, Tsutsui JM, Negrão CE, Canavesi N, Carvalho Frimm C, et al. Effects of exercise training on myocardial blood flow reserve in patients with heart failure and left ventricular systolic dysfunction. Am J Cardiol. 2010;105: 243–8. doi:10.1016/j.amjcard.2009.09.009

10. Position of the American Heart Association on research animal use. Circulation. 1985;71: 849A–50A. Available: http://www.ncbi.nlm.nih.gov/pubmed/3971549

11. Mattoso AAA, Kowatsch I, Tsutsui JM, de la Cruz VY, Ribeiro HB, Sbano JCN, et al. Prognostic value of qualitative and quantitative vasodilator stress myocardial perfusion echocardiography in patients with known or suspected coronary artery disease. J Am Soc Echocardiogr. 2013;26: 539–47. doi:10.1016/j.echo.2013.01.016

12. Peltier M, Vancraeynest D, Pasquet A, Ay T, Roelants V, D’hondt AM, et al. Assessment of the physiologic significance of coronary disease with dipyridamole real-time myocardial contrast echocardiography. Comparison with technetium-99m sestamibi single-photon emission computed tomography and quantitative coronary angiography. J Am Coll Cardiol. 2004;43: 257–64. Available: http://www.ncbi.nlm.nih.gov/pubmed/14736446

13. Dariolli R, Takimura CK, Campos C a, Lemos P a, Krieger JE. Development of a closed-artery catheter-based myocardial infarction in pigs using sponge and lidocaine hydrochloride infusion to prevent irreversible ventricular fibrillation. Physiol Rep. 2014;2: 1–11. doi:10.14814/phy2.12121

14. Schneider CA, Rasband WS, Eliceiri KW. NIH Image to ImageJ: 25 years of image analysis. Nat Methods. 2012;9: 671–5. Available: http://www.ncbi.nlm.nih.gov/pubmed/22930834

15. Malliaras K, Li T-S, Luthringer D, Terrovitis J, Cheng K, Chakravarty T, et al. Safety and efficacy of allogeneic cell therapy in infarcted rats transplanted with mismatched cardiosphere-derived cells. [Internet]. Circulation. 2012. doi:10.1161/CIRCULATIONAHA.111.042598

16. Stewart S, Winters GL, Fishbein MC, Tazelaar HD, Kobashigawa J, Abrams J, et al. Revision of the 1990 working formulation for the standardization of nomenclature in the diagnosis of heart rejection. J Heart Lung Transplant. 2005;24: 1710–20. doi:10.1016/j.healun.2005.03.019

17. Whittaker P, Kloner RA, Boughner DR, Pickering JG. Quantitative assessment of myocardial collagen with picrosirius red staining and circularly polarized light. Basic Res Cardiol. 1994;89: 397–410. doi:10.1007/BF00788278

18. Langen M, Peters U, Körner U, Gissel C, Stanislawski D, Klein G. Semiquantitative detection of male pork tissue in meat and meat products by PCR. MESC. The American Meat Science Association; 2010;86: 821–824. doi:10.1016/j.meatsci.2010.07.003
